# Effect of altered gluteus maximus strength on the magnitude and direction of hip joint contact forces during simulations of gait

**DOI:** 10.1371/journal.pone.0324451

**Published:** 2025-06-23

**Authors:** Jay R. Patel, Madeline Grosklos, Cara L. Lewis, Stephanie Di Stasi

**Affiliations:** 1 School of Health and Rehabilitation Sciences, The Ohio State University, Columbus, Ohio, United States of America; 2 Sports Medicine Research Institute, The Ohio State University Wexner Medical Center, Columbus, Ohio, United States of America; 3 Department of Biomedical Engineering, The Ohio State University, Columbus, Ohio, United States of America; 4 Department of Physical Therapy, Sargent College of Health & Rehabilitation Sciences, Boston University, Boston, Massachusetts, United States of America; 5 Division of Physical Therapy, School of Health and Rehabilitation Sciences, The Ohio State University, Columbus, Ohio, United States of America; University College Dublin, IRELAND

## Abstract

Femoroacetabular Impingement Syndrome (FAIS), a common and painful hip condition that affects active young adults, is associated with muscle weakness and altered movement patterns during common daily activities. FAIS is believed to be a precursor to osteoarthritis through disrupted loading patterns; thus, understanding how weakness affects joint loading may provide insights into the pathogenesis from FAIS to osteoarthritis. The aim of this study was to examine the impact of gluteus maximus strength on hip joint contact forces during gait using musculoskeletal modeling. Twelve individuals with FAIS and 13 healthy controls were included in the study and five conditions of gluteus maximus strength (50%, 75%, 100%, 125%, and 150% of the model’s default strength) were examined. Differences in the magnitude of the resultant joint contact force and directional components of the joint contact force between groups (FAIS vs controls) and between strength conditions were assessed with statistical nonparametric mapping. Joint contact forces were also used to determine the direction of hip loading in the sagittal and frontal planes and the center of the loading area was compared between groups and between strength conditions using independent t-tests and repeated measures analysis of variance, respectively. Joint contact force magnitude and direction did not differ between FAIS and control groups, aside from a brief period of larger medial force in the FAIS group. With both groups combined for remaining analyses, magnitude and direction of the joint contact force changed with the varied strength conditions; specifically, the 50 and 75% strength conditions demonstrated a lower magnitude joint contact force with a more anterior and medial direction. Importantly, these changes were small and of questionable clinical value. Future investigations should model movement changes alongside strength changes and use magnetic resonance imaging to examine a mechanistic link between load and intraarticular joint health.

## Introduction

Femoroacetabular Impingement Syndrome (FAIS) is a debilitating hip condition prevalent in active individuals in their second to fourth decades of life [1–3]. FAIS is defined by motion-related pain in the presence of atypical bony morphology of the femoral head and/or acetabular wall [3]. The morphologies associated with FAIS can cause premature contact of the femoral head on the acetabular wall and are theorized to instigate pain and soft tissue deterioration in the anterosuperior region of the acetabulum leading to osteoarthritis (OA) [4]. Rehabilitation for individuals with FAIS emphasizes strengthening of the extensor muscle group as a means to improve joint stability and lessen ailments of the condition, especially in those with strength deficits [5–9].

Hip extensor weakness of 10–25% has been identified pre- and post-arthroscopy in individuals with FAIS when compared to healthy controls [10,11]. Individuals with FAIS demonstrate slower contraction time in the gluteus maximus compared to their unaffected limb [12] and gluteus maximus atrophy as indicated by smaller cross-sectional area compared to controls [13]. Further, increased hip extensor strength has been correlated to improved patient reported function in FAIS [10,11,16].

The hip extensor muscles generate the greatest torque of all hip-spanning muscle groups [14], with the gluteus maximus being the largest and a potent contributor to motion in all three planes [14,15]. The extensors play an important role during both the swing and stance phases of walking: they decelerate the limb in preparation for contact during swing and provide vertical support, limb extension, and control of forward momentum during stance [17]. The gluteus maximus contributes a substantial portion of the total force in the first half of the gait cycle [15], extending the stance limb under the body at initial contact, stabilizing the pelvis, and aiding in trunk extension [17]. The size and functional importance of the gluteus maximus make it an appealing target for strengthening in rehabilitation [8,9].

Despite the focus on hip extensors in rehabilitation for FAIS [8,9], there is little information on the implications of extensor strength on joint contact forces (JCFs) within the hip. Musculoskeletal simulations in OpenSim can be used to evaluate the cause-and-effect relationships of gluteus maximus strength on hip joint loading during gait [18–21]. One simulation study found that the anterior component of the JCF increased under conditions of decreased gluteal muscle strength during prone hip extension [22], a common exercise in rehabilitation. While hip extensor weakness has been observed in individuals with FAIS compared to controls [10,11], simulation studies have failed to identify a difference in gluteus maximus force production during normal walking between groups [23–25]. An improved understanding of how gluteus maximus strength impacts JCFs during functional tasks such as gait is necessary to determine the most important contributors to altered joint loading in FAIS.

The primary aim of this study was to understand changes in magnitude and direction of hip JCFs during walking gait with varied levels of simulated gluteus maximus strength, including clinically relevant ranges of weakness observed in patients with FAIS [10,11]. We hypothesized that simulated modifications to gluteus maximus strength would change both the magnitude and direction of hip JCFs during gait. To account for potential baseline differences in gluteus maximus force production and JCFs between individuals with FAIS and healthy controls, we first compared these measures between groups. Based on findings from previous simulation investigations, we hypothesized that there would be no difference in gluteus maximus force production between groups [23–25], and that participants with FAIS would have lower magnitude [23,24,27] and more anteriorly directed contact forces [27] than healthy controls.

## Materials and methods

### Participants

All methods in the present study were approved by The Ohio State University institutional review board and written informed consent was obtained from all individuals prior to participation. Participants for this study were included in two additional simulation studies currently under review. Participants with FAIS and healthy controls between the ages of 16–40 years old were recruited from The Ohio State University Medical Center and the greater Columbus area from 22 July 2022–26 April 2023. Individuals with FAIS were required to have a unilateral FAIS diagnosis, no concomitant or previous hip conditions, no history of surgery to the involved hip, and no recent (<2 years) history of surgery to the involved lower extremity or spine. Healthy controls were required to have no concurrent or prior hip conditions, no hip pain in the week leading up to partaking in the study, and no recent (<2 years) history of surgery to the involved lower extremity or spine. Following informed consent, the flexion, adduction, and internal rotation (FADIR) test [3,28–30] was used to confirm proper inclusion (i.e., that the FAIS participants’ hip pain was joint-related and that healthy control participants did not present with pain). FAIS and healthy control participants who indicated a negative and positive FADIR, respectively, were removed from the study.

### Experimental data collection

Hip-related physical function and activity level were measured with the 33-item International Hip Outcome Tool (iHOT-33) [31] and Hip Sports Activity Scale (HSAS) [32], respectively. Overall self-efficacy and kinesiophobia were measured with the Pain Self-efficacy Questionnaire (PSEQ) [33] and 11-item Tampa Scale of Kinesiophobia (TSK-11) [34], respectively. Hip muscle strength was measured for all participants through maximal voluntary isometric contraction (MVIC) tests. MVIC trials were measured with a strap-fixated handheld dynamometer (Commander Echo, JTECH Medical Industries Inc., Midvale UT USA) for hip flexion, extension, adduction, abduction, internal rotation, external rotation, and knee flexion and extension. Participants were situated on a standard physical therapy treatment table and pushed against the device, secured to the testing table by a strap, as hard as possible while limiting trunk motion. Three trials of a five second isometric hold were performed for each strength test and the largest value was used for analysis. Torque was calculated for each strength test through multiplying the maximum force by thigh or shank length and subtracting a fixed distance to account for dynamometer placement (7 cm for thigh, 4 cm for shank).

For 3D motion capture, participants were fitted with 59 retroreflective markers in a modified Helen-Hayes arrangement [35] including rigid clusters on the shank and thigh [36] and a standard lab-issued Nike Pegasus shoe with markers embedded. Scale factors for modeling were determined via static capture of participant in a “T-pose”, standing with feet shoulder-width apart and arms abducted at 90 degrees. Participants completed over ground gait trials at a self-selected walking pace while marker trajectories and ground reaction forces (GRF) were recorded at 240 Hz and 1200 Hz respectively using a 12-camera motion capture system (Raptor, Motion Analysis Co., Santa Rose, CA, USA) and two floor-embedded force plates (Bertec Co. Worthington, OH, USA).

### Data processing and simulations

**Fig 1:**
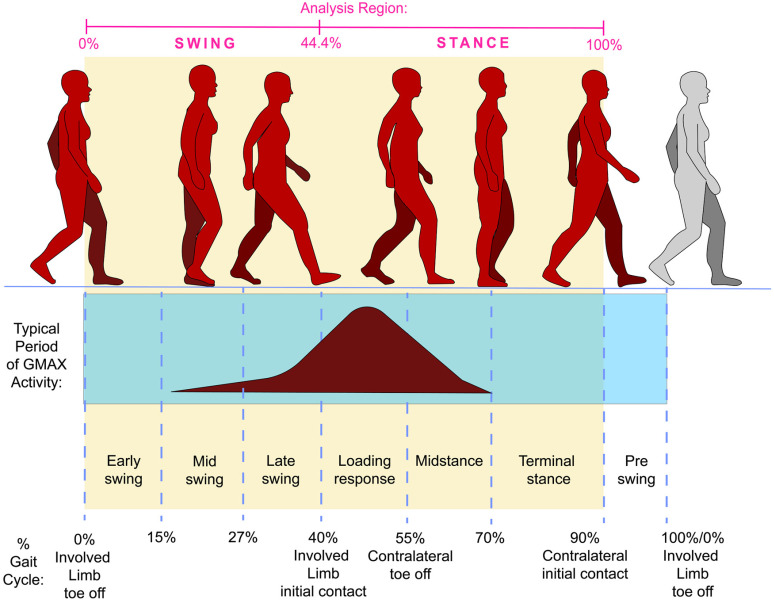
Demonstration of analysis region relative to the gait cycle. The analysis region was from toe off of the involved limb to contralateral heel strike and is shown in the shaded box. The timeline at the top of the figure shows analysis region percentages matched to important gait events (i.e., 0% of the analysis region occurs at 60% of the typical gait cycle, or at the time of involved limb toe-off). A full gait cycle was unable to be obtained due to limited ground reaction forces provided by the set-up of the force plates.

The first of the collected gait trials to demonstrate contact of the uninvolved limb on the first and involved limb on the second force plate was selected and analyzed from involved-limb toe off to contralateral-limb heel strike (i.e., swing through terminal stance of the involved limb for one stride) (Fig 1). This region of analysis captured the typical period of gluteus maximus activity, which begins during swing and continues into stance (Fig 1). Marker trajectories and GRF signals from the static trial and single gait trial were imported into Visual3D (C-Motion Inc. Rockville, MD, USA) for calculation of scale factors and inverse kinematics [37]. Gait kinematics and GRFs were then filtered with a second order low-pass bidirectional Butterworth filter at 6 Hz [38] and exported as OpenSim motion files. The OpenSim Gait2392 model [20] with Millard muscle dynamics [39] was scaled for each participant using participant mass and scale factors exported from Visual3D designed specifically for use with the Gait2392 model [37]. Subtalar and metatarsophalangeal joint degrees of freedom were locked in the model [49].

**Fig 2:**
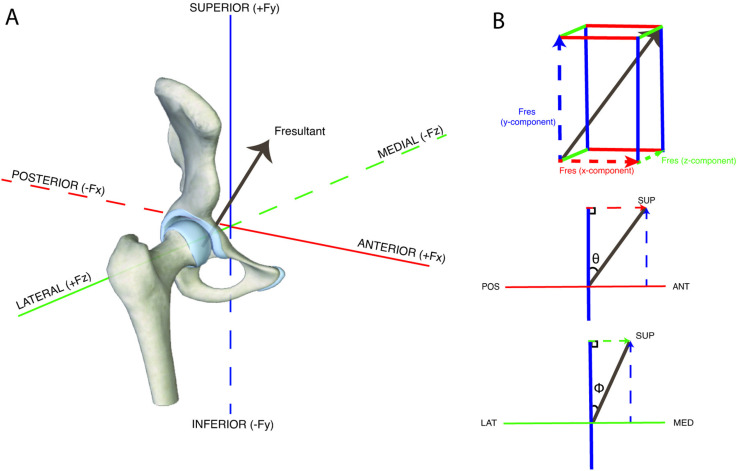
A: Diagram of femur on acetabulum resultant joint contact forces (JCFs) in the pelvic reference frame. B: Assistive diagram showing proposed variables: theta (Θ) and phi (Φ) in respective right-triangles formed in a planar (2D) view. Fres = Resultant JCF; POS = posterior; ANT = anterior; LAT = lateral; MED = medial; SUP = superior.

Within OpenSim, the residual reduction algorithm (RRA) was used to improve consistency between model mass, kinematics, and GRF data [20]. RRA was iterated until overall model mass change was < 0.001 kg [40], root-mean-square and maximum residual forces and moments were less than or as close as possible to 5 and 10N, and 30 and 50Nm, respectively, and kinematic errors were less than or as close as possible to 2° for joint rotations and 2 cm for pelvis translations [20,41]. The scaled, RRA-adjusted model was saved with five conditions of modified gluteus maximus strength: 50%, 75%, 100%, 125%, and 150% of the default model’s maximum isometric force production value. Computed muscle control (CMC) [42,43] and the joint reaction analysis tool [44] were run on each of the five conditions to estimate muscle force production and JCFs, respectively. CMC was performed with a 2ms look-ahead window rather than the OpenSim default of 10ms. Based on pilot work, this was necessary to improve kinematic tracking and reduce kinematic errors, specifically at the ankle. JCFs were calculated as femur-on-acetabulum forces and presented in the pelvic reference frame (Fig 2A). Simulations were validated through comparisons to electromyography signals and quality controlled at each simulation step (S2 Appendix). Activation and force production of all hip and thigh muscles were exported for analysis and visualization (S3 Appendix, S4 Appendix). JCFs and gluteus maximus muscle force production were normalized to body weight (BW) for comparisons between groups and strength conditions.

### Joint contact force direction

Direction of JCFs was assessed through calculation of theta (Θ) and phi (Φ) angles from the three planar components of the JCF. Theta represented the angle of the resultant JCF in the sagittal plane (i.e., the angle between the frontal plane and the resultant vector) (Fig 2B). Positive values for Θ indicated an anteriorly directed JCF and negative values for Θ indicated a posteriorly directed JCF. Phi represented the angle of the resultant JCF in the frontal plane (i.e., the angle between the sagittal plane and the resultant vector) (Fig 2B). Θ vs Φ plots were created to visualize the vector path in the transverse plane. A MATLAB algorithm was developed using the MATLAB Optimization Toolbox to perform a least-square regression to fit an elliptical equation to the vector path. From this equation, the center of the Θ vs Φ path was extracted and represented the regional loading centers of the JCFs.

### Statistical analysis

For the timeseries analysis of gluteus maximus force production and JCF magnitude, statistical nonparametric mapping (SnPM) [45–48] was performed using the opensource spm1d code (v.M.0.4.11, www.spm1d.org) in MATLAB. All discrete variable analyses including demographic comparison, hip muscle strength, and comparison of regional loading centers were performed in SPSS Statistics (v29.0, IBM Corp. Armonk, NY). Differences in time series curves for gluteus maximus force production and directional JCF components (anterior, superior, medial) between groups were assessed with an independent samples SnPM Hotelling’s T^2^ test. Differences in time series curves of resultant JCFs between groups were assessed with an SnPM two-tailed independent t-test. Regional loading centers were compared between groups with a two-tailed independent samples t-test. To aid in interpreting results, angles and moments at the hip, knee, and ankle were compared between groups using SnPM two-tailed independent samples t-tests.

Differences in the resultant JCF and directional components of the JCF between gluteus maximus strength conditions (50%, 75%, 100%, 125%, 150%) were assessed with SnPM repeated measures analysis of variance (rmANOVA) and Hotelling’s T^2^ tests, respectively. Differences in the estimated direction of the resultant JCF between gluteus maximus strength conditions were assessed with separate rmANOVAs for the medial-lateral and anterior-posterior position of the regional loading center under each strength condition. If significance was found in either the Hotelling’s T^2^ or rmANOVA tests, post hoc independent (between groups) or paired (between strength conditions) t-tests with custom contrasts were performed to compare each modified gluteus maximus strength condition to the default (100%) condition. To aid the interpretation of the directional JCF data, 3D plots were generated to visualize how the direction of the JCF components varied under different strength conditions. No statistical analysis of these plots was performed.

## Results

A total of 28 participants were considered for this study and 25 participants were retained in analysis, 12 with FAIS (8F, 4M) and 13 healthy controls (7F, 6M). One FAIS participant was removed due to marker placement errors on the pelvis, and one FAIS and one healthy control participant were removed due to simulation failure during CMC. No differences were found between groups for age, height, or BMI (p ≥ 0.123). The FAIS group had lower body mass (p = .046) and walked significantly slower (p = .035) than the healthy control group. The FAIS group also had lower PSEQ and iHOT scores (p < .001), indicating worse self-efficacy [33] and hip-related function [31], respectively and a higher TSK-11 score than the healthy control group (p < .001), indicating greater kinesiophobia [34] (Table 1). There were no differences in the hip muscle strength of the involved limb (p ≥ .098) (Table 2) or biomechanics, including sagittal plane moments and angles for the hip, knee, and ankle, and frontal plane moment and angle for the hip, between FAIS and healthy control groups (S1 Appendix).

**Table 1:** Demographic representation of participants in the FAIS and healthy control groups. Data were reported as mean ± standard deviation for continuous measures and median (interquartile range) otherwise.

	FAIS	Controls	P-value
N (F, M)	12 (8F, 4M)	13 (7F, 6M)	
Age	29.1 ± 6.3	28.2 ± 7.2	.728
Height (m)	1.70 ± 0.08	1.74 ± 0.10	.347
Mass (kg)	64.5 ± 10.6	73.2 ± 12.3	.046
BMI	22.3 ± 2.5	24.2 ± 3.1	.123
PSEQ	48.9 ± 9.1	59.5 ± 1.9	<.001
iHOT	61.1 ± 20.5	98.9 ± 1.4	<.001
TSK-11	23.2 ± 4.4	14.1 ± 2.6	<.001
Highest activity prior to symptoms	5 (2)	3 (2)	.183
Current activity	3 (2)	3 (2)	.265
Gait speed (m/s)	1.26 ± 0.07	1.40 ± 0.19	.035

**Table 2:** Maximum involved limb strength values normalized to body mass and presented as measured force (N/kg) and torque (Nm/kg) for individuals with femoroacetabular impingement syndrome (FAIS) and healthy controls. Data are presented as median (interquartile range).

Task	Force (N/kg)			Torque (Nm/kg)		
	FAIS	Controls	p-value	FAIS	Controls	p-value
Hip Extension	2.21 (1.19)	2.78 (1.50)	.137	0.81 (0.44)	1.01 (0.60)	.152
Hip Flexion	2.24 (1.01)	2.09 (0.40)	.979	0.85 (0.40)	0.77 (0.15)	1.000
Hip Adduction	2.52 (1.04)	2.92 (1.13)	.270	0.90 (0.43)	1.09 (0.41)	.168
Hip Abduction	3.36 (0.85)	3.88 (1.11)	.098	1.20 (0.38)	1.44 (0.56)	.123
Hip Internal Rotation	1.44 (1.05)	1.47 (0.67)	.689	0.46 (0.36)	0.48 (0.19)	.538
Hip External Rotation	1.19 (0.67)	1.25 (0.56)	.437	0.38 (0.21)	0.43 (0.18)	.270
Knee Extension	3.12 (0.97)	3.11 (1.20)	.574	1.07 (0.31)	1.14 (0.48)	.320
Knee Flexion	1.89 (0.81)	2.09 (0.76)	.437	0.63 (0.30)	1.70 (0.31)	.376

All simulations met OpenSim quality standards for RRA and CMC residual forces and moments and kinematic errors [49]. All but two simulations met OpenSim-recommended standards for peak reserve actuator torque; these two participants exceeded recommended torque thresholds at the ankle joint, were the two tallest participants, and were in the 85^th^ percentile for gait speed (i.e., two of the fastest walkers in the group). Simulations were validated through qualitative comparison to experimental EMG (S2 Appendix Figs S2.1–S2.4) [41].

Changes in gluteus maximus strength caused changes to activation and force production of numerous muscles of the hip and thigh (S3 Appendix, S4 Appendix). Between individuals with FAIS and healthy controls, there were no differences found in gluteus maximus force production (S5 Appendix, Figs S5.1 A-C), resultant JCFs (S5 Appendix, Fig S5.2), anterior and superior components of the JCF (S5 Appendix, Fig S5.3 A-D), or regional loading centers during gait (S5 Appendix, Fig S5.4). Individuals with FAIS demonstrated larger medial JCFs than healthy controls from 65–71% of the analysis region (p = .003) (S5 Appendix, Fig S5.3 E and F).

**Fig 3:**
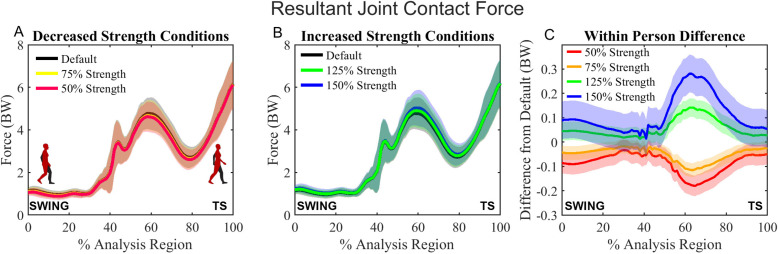
A: Timeseries curves for the resultant joint contact force (JCF) of the default, 50% and 75% conditions; B: Timeseries curves for the resultant joint contact force (JCF) of the default, 125%, and 150% conditions; C: Differences in JCF magnitude between each scaled condition and the default. Analysis region begins at swing and ends at terminal stance (TS) as indicated on the x-axis.

Participants from the FAIS and healthy control groups were combined to test the effects of gluteus maximus strength on magnitude and direction of JCFs. For the resultant JCF magnitude, SnPM rmANOVA revealed significant differences between the gluteus maximus strength conditions through the entire analysis region (p = .001) (S5 Appendix, Fig S5.5). Post hoc t-tests revealed that the resultant JCF in the 50% and 75% strength conditions was lower than that of the default condition throughout a majority of the analysis region; however, the difference in magnitude from default was only 0.086 ± 0.043BW for the 50% condition and 0.050 ± 0.029BW for the 75% condition (Fig 3, Table 3, S5 Appendix, Fig S5.6). For the 125% and 150% conditions, the resultant JCF was higher than that of the default condition for a majority of the analysis region, and the difference in magnitude compared to default was 0.056 ± 0.038BW for the 125% condition and 0.113 ± 0.078BW for the 150% condition (Fig 3, Table 3, S5 Appendix, Fig S5.6).

**Table 3:** Post hoc SnPM paired t-test results for the resultant joint contact force (JCF) and the average of the difference between conditions across the analysis region. Direction of difference compares each strength condition to default. Significant differences with the specified p-value occurred over the listed range of the analysis region.

Condition	Direction of difference	P-value	Significant region (s) (% Analysis region)	Average (±SD) of the difference (BW)
50% vs Default	Decrease	<.001	0-41, 43-100	−.086 ± .043
75% vs Default	Decrease	<.001	0-37, 39-41, 44-100	−.050 ± .029
125% vs Default	Increase	<.001	0-38, 39-40, 43-100	.056 ± .038
150% vs Default	Increase	<.001	0-38, 39-40, 42-100	.113 ± .078

**Fig 4:**
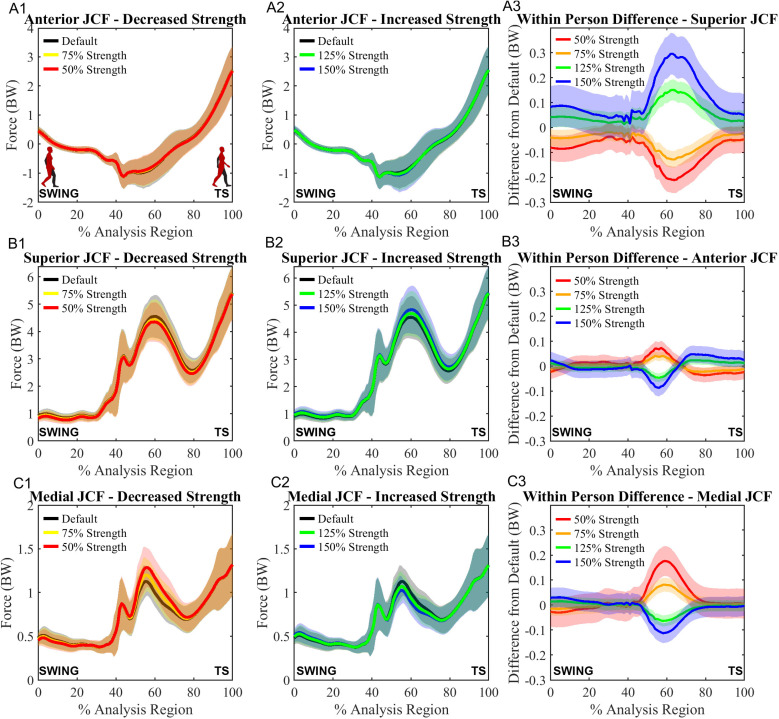
Timeseries curves for the anterior, superior, and medial components of the joint contact force (JCF) for the default, 50% and 75% conditions (column 1), the default, 125%, and 150% conditions (column 2), and the difference in JCF magnitude between each scaled condition and the default (column 3). A1-3: Data for the anterior JCF. B1-3: Data for the superior JCF. C1-3: Data for the medial JCF. Analysis region begins at swing and ends at terminal stance (TS) as indicated on the x-axis.

For the directional components of the JCF, SnPM vector field Hotelling’s T^2^ tests revealed differences in force magnitude between the five strength conditions (p = .001). The superior JCF was the only component to demonstrate a change that was maintained throughout the analysis region (Table 4). Compared to the default condition, the decreased strength conditions demonstrated lower superior JCF and the increased strength conditions demonstrated higher superior JCF for most of the analysis region (p < .001) (Table 4). The anterior (p ≤ .001) and medial (p ≤ .001) components of the JCF demonstrated inconsistent changes under all conditions compared to default (Table 4, Figs 4A3 and 4C3). Under decreased strength conditions, the anterior component decreased early in the analysis region, followed by a region of increase, then another decrease late in the analysis region (Table 4, S5 Appendix Fig S5.6 A). The opposite trend was observed for the increased strength conditions; increase in force, followed by decrease, then a final increase late in the analysis region (Table 4, S5 Appendix Fig S5.6 B). For the medial component, decreased gluteus maximus strength caused a decrease in force in the early half of the analysis region and an increase in force in the latter half (Table 4, S5 Appendix Fig S5.6 E). Again, the opposite was observed for increased strength conditions with increased medial JCF in the early half of the analysis region and decreased medial JCF in the latter half (Table 4, S5 Appendix Fig S5.6 F).

**Table 4:** Post hoc SnPM paired t-test results for the anterior, superior, and medial components of the joint contact force (JCF). Direction of difference compares each strength condition to default. Significant differences with the specified p-value occurred over the listed range of the analysis region.

Component	Condition	Direction of difference	P-value	Significant region (s) (% Analysis region)
Anterior	50% - Default	Decrease	p < .001	0-4, 74-99
		Increase	p < .001	11-40, 45-49, 52-59
	75% - Default	Decrease	p < .001	0-4, 73-98
		Increase	p ≤ .001	12-33, 35-37, 47-49, 52-59
	125% - Default	Increase	p < .001	0-3, 71-99
		Decrease	p ≤ .001	11-37, 46-47, 49-58
	150% - Default	Increase	p < .001	0-3, 71-99
		Decrease	p < .001	11-37, 46-47, 49-58
Superior	50% - Default	Decrease	p < .001	0-41, 43-100
	75% - Default	Decrease	p < .001	0-37, 39-40, 44-199
	125% - Default	Increase	p < .001	0-37, 39-40, 43-100
	150% - Default	Increase	p < .001	0-37, 39-40, 42-100
Medial	50% - Default	Decrease	p < .001	0-24
		Increase	p ≤ .002	47-77
	75% - Default	Decrease	p < .001	0-25
		Increase	p < .001	48-76
	125% - Default	Increase	p < .001	0-25, 36-37
		Decrease	p < .001	48-78, 93-95
	150% - Default	Increase	p < .001	0-26, 34-37, 39
		Decrease	p ≤ .001	48-76, 91, 94-95

**Fig 5:**
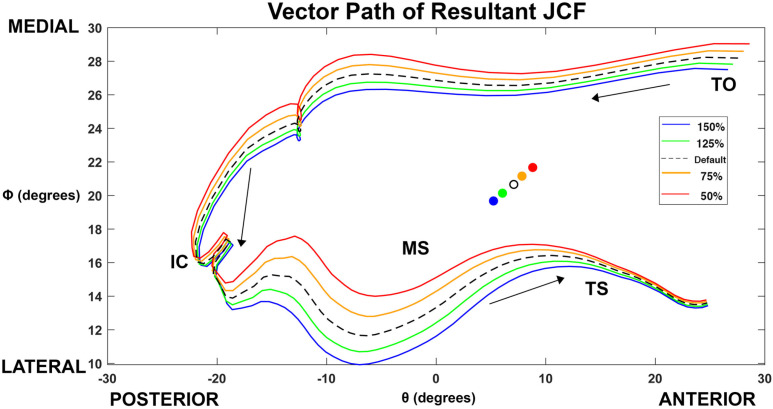
Theta (Θ) vs Phi (Φ) joint contact force (JCF) vector path of increased and decreased strength conditions in comparison to default condition (100%). Gait event labels are placed for orientation at beginning and end of vector path: TO = involved toe off (start of the analysis region), IC = initial contact, MS = midstance, TS = terminal stance (end of the analysis region). Color-coordinated circles represent regional loading centers of each condition calculated per least squares algorithm.

Visual assessment of three-dimensional force vectors revealed the greatest difference in the JCF between conditions during loading response and midstance, around the time of contralateral toe-off (Fig 5). The 50% strength condition demonstrated a visible shift towards the medial, inferior, and anterior directions (Fig 5A), while the 150% condition demonstrated a shift towards lateral, superior, and posterior directions (Fig 5D).

**Fig 6:**
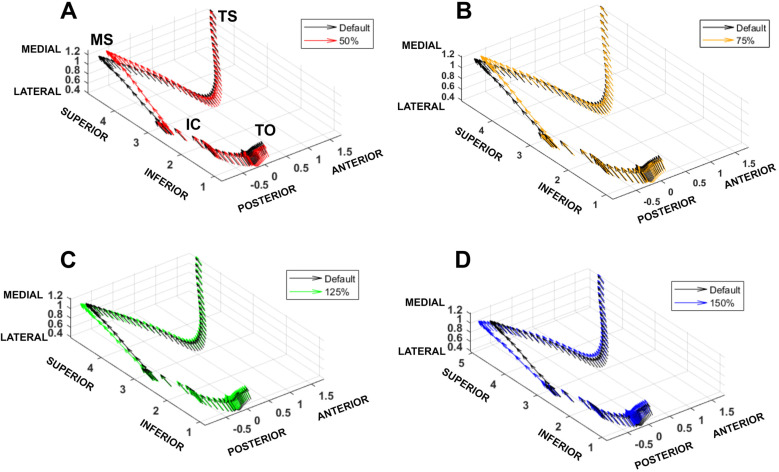
3D plots comparing resultant joint contact force (JCF) magnitude (Force [BW]) between the default and each strength condition. Gait cycle events are labeled in A and correspond to all plots. A: 50% vs default (100%) condition. B: 75% vs default condition. C: 125% vs default condition. D: 150% vs default condition. TO = involved toe off, IC = involved initial contact, MS = involved midstance, TS = involved terminal stance.

As gluteus maximus strength increased from 50% to 150%, the regional loading center shifted posteriorly and laterally, with significant differences in at least one direction for all conditions compared to default (p ≥ .035) (Fig 6). Theta (location in the anterior-posterior direction) was larger, indicating a more anterior JCF direction, for the 50% condition compared to default (mean difference = 1.73°, p = .035) and smaller, indicating a less anterior JCF direction, for the 125% and 150% conditions compared to default (mean difference = −1.03°, p < .001; mean difference = −1.85°, p < .001, respectively). Phi (location in the medial-lateral direction) was larger, indicating a more medial JCF direction, for the 50% and 75% conditions compared to default (mean difference = 1.01°, p < .001; mean difference = 0.50°, p < .001, respectively) and smaller, indicating a less medial JCF direction, for the 125% and 150% conditions compared to default (mean difference = −0.52°, p < .001; mean difference = −0.99°, p < .001, respectively). Visual assessment of the JCF paths in the transverse plane indicate a shift away from the medial direction (i.e., decreasing Φ angle) during swing and midstance under increased gluteus maximus strength conditions (Fig 6).

## Discussion

The aims of this study were to: 1) compare estimated gluteus maximus force production and JCF magnitude and direction during gait between individuals with FAIS and healthy controls, and 2) explore the effects of simulated decreased and increased gluteus maximus strength on the magnitude and direction of JCFs during gait. We observed minimal differences in gluteus maximus force production and JCF magnitude or direction between FAIS and healthy control groups. Both magnitude and direction of hip JCFs changed with varied gluteus maximus strength conditions; the clinical impact of the changes observed in this cross-sectional study are unclear.

The lack of differences in gluteus maximus force production and resultant JCF magnitude between the FAIS and healthy control groups is consistent with the lack of group differences in involved limb hip muscle strength and biomechanics in the present cohort. For gluteus maximus force production, our findings are also consistent with three previous investigations [23–25], suggesting that individuals with FAIS may not have altered gluteus maximus function during gait that is observable through musculoskeletal modeling. Further, as gait does not typically require near-maximal force production from the gluteus maximus muscle, it is unlikely that moderate amounts of real-world gluteus maximus weakness in individuals with FAIS would result in movement alterations that the simulation translates to force production differences during this task, which could explain the lack of difference seen in previous works that inspired the hypothesis for this study. However, the lack of difference in resultant JCF magnitude disagrees with three previous studies that found lower JCFs during gait in individuals with FAIS compared to healthy controls [23,24,26,27]. Two of these studies found decreased internal hip flexion moments and increased hip adduction angles during terminal stance [23,24], potentially contributing to the observed differences in resultant JCF magnitude not seen in this study. The lack of difference in JCF direction also disagrees with one previous study which found lateral and anterior shifts in JCF vector localization in individuals with FAIS compared to healthy controls [27]. The lack of agreement may be related to demographic differences in the studies. Two of the studies investigated only male participants [23,24] while our work included both male and female participants. Further, in at least two of the studies, participants were either awaiting surgery at the time of data collection [24] or enrolled in a clinical trial for which the surgeon believed all participants would benefit from arthroscopic surgery [27]. In the present study, participants were not required to be awaiting surgery; it is possible that the participants in this study had less severe symptoms compared to those in previously published work, although none of the published studies used symptom scores that could be directly compared to our cohort.

The lack of differences in gluteus maximus force production and resultant JCF magnitude between the FAIS and healthy control groups is consistent with the lack of group differences in involved limb hip muscle strength and biomechanics in the present cohort. For gluteus maximus force production, our findings are also consistent with three previous investigations [23–25], suggesting that individuals with FAIS may not have altered gluteus maximus function during gait that is observable through musculoskeletal modeling. Further, as gait does not typically require near-maximal force production from the gluteus maximus muscle, it is unlikely that moderate amounts of real-world gluteus maximus weakness in individuals with FAIS would result in movement alterations that the simulation translates to force production differences during this task, which could explain the lack of difference seen in previous works that inspired the hypothesis for this study. However, the lack of difference in resultant JCF magnitude disagrees with three previous studies that found lower JCFs during gait in individuals with FAIS compared to healthy controls [23,24,26,27]. Two of these studies found decreased internal hip flexion moments and increased hip adduction angles during terminal stance [23,24], potentially contributing to the observed differences in resultant JCF magnitude not seen in this study. The lack of difference in JCF direction also disagrees with one previous study which found lateral and anterior shifts in JCF vector localization in individuals with FAIS compared to healthy controls [27]. The lack of agreement may be related to demographic differences in the studies. Two of the studies investigated only male participants [23,24] while our work included both male and female participants. Further, in at least two of the studies, participants were either awaiting surgery at the time of data collection [24] or enrolled in a clinical trial for which the surgeon believed all participants would benefit from arthroscopic surgery [27]. In the present study, participants were not required to be awaiting surgery; it is possible that the participants in this study had less severe symptoms compared to those in previously published work, although none of the published studies used symptom scores that could be directly compared to our cohort.

The change in magnitude of JCFs under varied conditions of gluteus maximus strength was small and of questionable clinical value. This could be a result of modeling strength changes while keeping kinematics constant. A 50% decrease in gluteus maximus strength only resulted in a mean difference of 8% bodyweight for the resultant JCF, indicating that despite being a primary contributor to the superior component of hip JCF during gait [50,51], changes in gluteus maximus strength without changes in movement may have little impact on the magnitudes of hip JCFs during gait. Thus, in patients with FAIS, weakness of the gluteus maximus, or strengthening of the gluteus maximus through rehabilitation without addressing movement patterns, may not cause any meaningful change to the magnitude of JCFs experienced at the hip during walking.

Our findings are similar to that of previous work that found normal gait appeared robust to gluteus maximus and vasti weakness [52]. Van der Krogt and colleagues found that 80% gluteus maximus weakness resulted in only a 10% increase in total lower extremity muscle stress. In comparison, 80% iliopsoas weakness resulted in a 135% increase in total muscle stress [52]. This indicates that weakness of the gluteus maximus is either minimally impactful or more easily compensated for during gait by surrounding musculature. A study simulating anterior JCFs with muscle weakness during common rehabilitation exercises showed that the effect of iliopsoas weakness may be more pronounced that that of gluteal weakness; weakened gluteal muscles (maximus, medius, and minimus) during prone hip extension led to a 39.5-unit increase in the total force contribution of muscle groups, while in comparison, weakened iliacus and psoas muscles during supine hip flexion resulted in a 140.4-unit increase [53]. Our finding of minimal JCF changes during gait with altered gluteus maximus strength, along with these previous studies, indicate that other muscle groups of the hip, such as the iliopsoas, may be more critical to restoring healthy loading magnitudes during walking in individuals with FAIS.

Gluteus maximus weakness during gait may have a more salient effect on the direction, rather than magnitude, of JCFs. Visual inspection of the 3D and vector path plots indicated a more anterior and medial direction of the JCF with decreased gluteus maximus strength. This finding is relevant as the anterosuperior region of the acetabulum is the primary location of tissue deterioration in FAIS [1,2,54,55]; disruption of the load to this vulnerable area may be relevant to symptoms experienced by these individuals. Investigation of cartilage and labral contact areas and strain via dual fluoroscopy and soft tissue modeling approaches found greater peak strain in the anterior labrum during midstance of gait and in the anterior cartilage during mid-swing of gait in individuals with FAIS compared to the control group during incline walking trials [56]. In our investigation, during both midstance and mid-swing, an anterior and medial shift of the JCF was observed in the decreased strength conditions, indicating the vector path moved towards the proposed area of pain and tissue deterioration [1,2,54,55]. The opposite pattern was observed in the increased strength conditions; during both midstance and mid-swing, the vector path moved posteriorly and laterally in the acetabulum. These results indicate that gluteus maximus weakness may contribute to increased concentration of loading in regions of joint damage in FAIS.

To our knowledge, this work is the first to investigate simulated magnitude and regional loading of JCFs during walking under varied conditions of hip muscle strength. As FAIS is recognized as an initial stage in the progression of hip OA [4], understanding both magnitude and direction of loading has the potential to inform load-altering treatments that may prevent joint degeneration in these patients. Alterations in intra-articular loading patterns are believed to contribute to the pathogenesis of knee OA [57], suggesting that both magnitude and regional loading of JCFs serve as important variables of consideration for rehabilitation [58,59]. Direction of loading may play a crucial role in the development of knee OA; similarly, studying regional loading patterns in FAIS should be encouraged to aid in identifying potential contributors to hip joint degeneration [60].

Limitations of this study include the limited sample size; as this was an exploratory study, an *a priori* power analysis was not conducted. Within OpenSim, kinematics were constrained to observed motion; thus, the simulations did not account for adjusted kinematics that may occur with differences in muscle strength. This study only observed the effects of gluteus maximus strength, and future work should investigate the effect of strength changes to the other gluteals and members of the extensor group. The generic Gait2392 model was not changed for hip-specific anatomy or gender differences. It is possible that differences between groups may have emerged had person-specific bone geometries been applied with more advanced modeling frameworks designed to estimate contact location. Additionally, the default strength parameters in the model may overestimate participant strength, as they are based upon data from a limited number of young male volunteers [61]. The experimental setup in this study prohibited analysis of a full gait cycle and future simulation studies should ensure the presence of at least 3 consecutive force plates for overground gait data collection. This work only analyzed walking gait, and it is possible that gluteus maximus strength plays a larger role in activities such as squatting, running, cutting, and jumping, all very relevant activities for young, active individuals with FAIS. Future work should build upon this investigation to understand the effect of gluteus maximus strength on JCFs during higher intensity activities.

This investigation revealed that the magnitude of hip JCFs during walking gait may not change substantially with varied gluteus maximus strength; however, decreased gluteus maximus strength did shift the regional loading medially and anteriorly. This finding indicates that addressing weakness of the gluteus maximus muscle in rehabilitation for FAIS may have a benefit in shifting forces away from the anterosuperior region, a common area of intra-articular damage, while the magnitude of loading may be less affected by strengthening protocols. Future work should aim to examine how weakness of other muscle groups, and other factors such as movement patterns, affect the magnitude and direction of hip joint load to examine targets for intervention. Similar types of investigations for other muscle groups of the hip will aid in informing data-driven approaches to focused muscle strengthening in patients with FAIS. While global strengthening of all hip musculature is assumed important, current practices that focus solely on improving hip extensor strength at the expense of strengthening other supporting musculature or modifying movement may not improve hip joint loads.

## Supporting information

S1 Appendix Biomechanics. (DOCX)

S2 Appendix Quality Control. (DOCX)

S3 Appendix Extensor Muscle Forces. (DOCX)

S4 Appendix Change in Activations and Forces. (DOCX)

S5 Appendix Additional Results. (DOCX)

S6 README README File for Manuscript Data and Code. (MD)

S7 Data Raw Gait Data for Publication 1 (Controls). (ZIP)

S8 Data Raw Gait Data for Publication 2 (FAIS). (ZIP)

S9 Data Full Participant Demographics. (XLSX)

S10 Code Matlab Code for Joint Force Direction Estimation. (ZIP)

S11 Files Visual3D Files and Models for Processing. (ZIP)
