# Correction: Effect of altered gluteus maximus strength on the magnitude and direction of hip joint contact forces during simulations of gait

**DOI:** 10.1371/journal.pone.0338414

**Published:** 2025-12-09

**Authors:** Jay R. Patel, Madeline Grosklos, Cara L. Lewis, Stephanie Di Stasi

The second author, Madeline Grosklos, is incorrectly noted as the corresponding author. The correct corresponding author is Stephanie Di Stasi. Stephanie Di Stasi’s email address is: distasi.2@osu.edu.
