# Supplementary material for: Effect of altered gluteus maximus strength on the magnitude and direction of hip joint contact forces during simulations of gait
Source: PLoS One. 2025 Jun 23;20(6):e0324451. doi: 10.1371/journal.pone.0324451 (PMC12184943; doi:10.1371/journal.pone.0324451)
Supplement: S1 Appendix — (DOCX) [file pone.0324451.s001.docx]

S1 Appendix: Angle and moment time series curves for the FAIS and healthy control groups


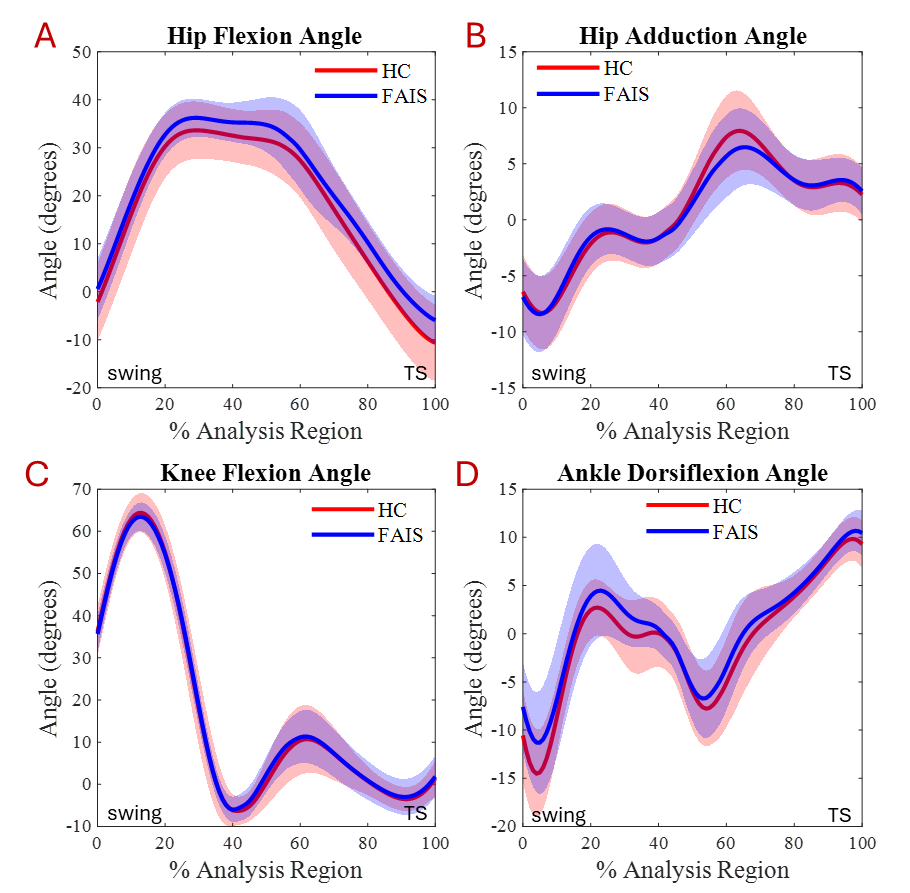


Figure S1.1: Hip, knee, and ankle angles over the analysis region, starting at involved limb toe off (marked by swing on the plot) and ending at contralateral foot strike (marked by TS = terminal stance on the plot), for participants with femoroacetabular impingement syndrome (FAIS) and healthy controls (HC). Counterclockwise: Hip flexion angle (A), Hip adduction angle (B), knee flexion angle (C), and ankle dorsiflexion angle (D). Statistical nonparametric mapping independent samples t-tests were run on all angles and no differences were found.


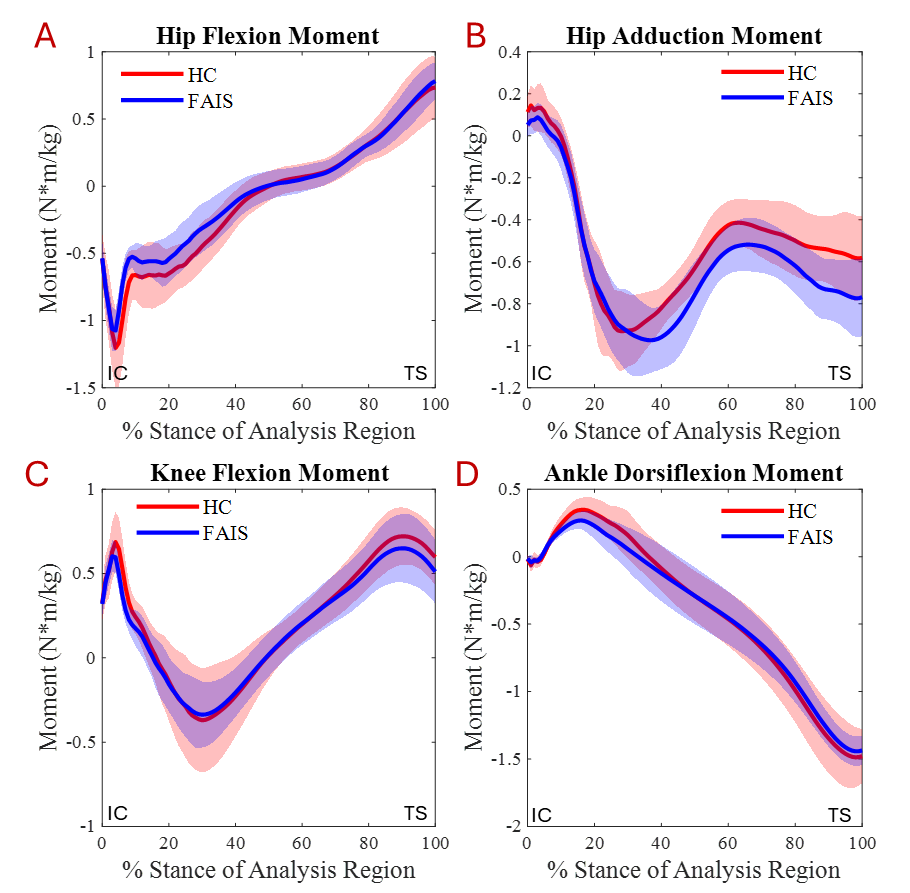


Figure S1.2: Hip, knee, and ankle moments (normalized to body mass) over stance phase of the analysis region, starting at involved limb foot strike (IC = initial contact on plot) and ending at contralateral foot strike (TS = terminal stance on plot), for participants with femoroacetabular impingement syndrome (FAIS) and healthy controls (HC). Counterclockwise: Hip flexion moment (A), Hip adduction moment (B), knee flexion moment (C), and ankle dorsiflexion moment (D). Statistical nonparametric mapping independent samples t-tests were run on all moments and no differences were found.
