# Supplementary material for: Effect of altered gluteus maximus strength on the magnitude and direction of hip joint contact forces during simulations of gait
Source: PLoS One. 2025 Jun 23;20(6):e0324451. doi: 10.1371/journal.pone.0324451 (PMC12184943; doi:10.1371/journal.pone.0324451)
Supplement: S2 Appendix — (DOCX) [file pone.0324451.s002.docx]

S2 Appendix: Quality Control

Table S2.1: Quality control results after running residual reduction algorithm (RRA) in OpenSim. RRA was iterated until all simulations met or were as close as possible to the recommended thresholds per OpenSim documentation of 0-10N for max residual force, 0-50Nm for max residual moment 0-5N for root-mean-square (RMS) residual force, 0-30Nm for RMS residual moment, 0-2cm for max and RMS kinematic error (pErr) for pelvic translations, and 0-2 degrees for max and RMS kinematic error (pErr) for joint rotations [1].

|  | Mean ± SD | Median (Q1, Q3) |
| --- | --- | --- |
| MAX Residual Force (N) | 7.20 ± 2.18 | 7.71 (5.43, 9.27) |
| MAX Residual Moment (Nm) | 27.35 ± 13.7 | 27.21 (13.98, 39.27) |
| RMS Residual Force (N) | 2.53 ± 0.92 | 2.39 (1.84, 3.08) |
| RMS Residual Moment (Nm) | 9.32 ± 3.81 | 10.47 (5.52, 12.26) |
| MAX kinematic error (cm) | 1.27 ± 0.53 | 1.13 (0.77, 1.61) |
| MAX kinematic error (deg.) | 0.22 ± 0.28 | 0.10 (0.04, 0.26) |
| RMS kinematic error (cm) | 0.75 ± 0.40 | 0.68 (0.43, 1.04) |
| RMS kinematic error (deg.) | 0.11 ± 0.14 | 0.05 (0.2, 0.12) |

Table S2.2: Quality control results after running computed muscle control (CMC) in OpenSim for all model configurations. All simulations met or were as close as possible to the recommended thresholds per OpenSim documentation of 0-10N for max residual force, 0-10N for root-mean-square (RMS) residual force, 0-50Nm for max residual moment, 0-30Nm for RMS residual moment, 0-1cm for max and RMS kinematic error (pErr) for pelvic translations, 0-2 degrees for max and RMS kinematic error (pErr) for joint rotations, 0-25Nm for max standard Gait2392 model reserve actuator torques, and 0-10Nm for RMS standard Gait2392 model reserve actuator torques. It is also recommended that peak reserve actuator torques should typically be less than 10% of the peak joint torque; this condition was more difficult to meet [1]. Kinematic errors and joint reserve data include average values across the pelvis (kinematics only), hip*, knee, and ankle joints. *Peak-Joint-Reserve-to-Peak-Joint-Torque Ratio includes average values for the frontal and sagittal plane only for the hip due to limitations of measuring moments in the transverse plane.

|  |  | 50% Strength | 75% Strength | 100% Strength | 125% Strength | 150% Strength |
| --- | --- | --- | --- | --- | --- | --- |
| MAX Residual Force (N) | Mean ± SD | 7.85 ± 2.64 | 7.85 ± 2.63 | 7.85 ± 2.65 | 7.85 ± 2.63 | 7.85 ± 2.63 |
|  | Median (Q1, Q3) | 8.52 (5.78, 10.04) | 8.50 (5.79, 10.01) | 8.52 (5.78, 10.04) | 8.52 (5.79, 10.04) | 8.52 (5.79, 10.04) |
| MAX Residual Moment (Nm) | Mean ± SD | 27.31 ± 14.00 | 27.31 ± 14.01 | 27.32 ± 14.04 | 27.34 ± 14.02 | 27.33 ± 14.01 |
|  | Median (Q1, Q3) | 26.96 (12.75, 39.23) | 26.96 (12.67, 39.29) | 26.96 (12.55, 39.25) | 26.96 (12.78, 39.27) | 26.96 (12.77, 39.26) |
| RMS Residual Force (N) | Mean ± SD | 2.58 ± 0.95 | 2.58 ± 0.95 | 2.58 ± 0.95 | 2.58 ± 0.95 | 2.58 ± 0.95 |
|  | Median (Q1, Q3) | 2.42 (1.88, 3.06) | 2.42 (1.88, 3.07) | 2.42 (1.88, 3.07) | 2.42 (1.88, 3.07) | 2.42 (1.88, 3.07) |
| RMS Residual Moment (Nm) | Mean ± SD | 9.33 ± 3.88 | 9.33 ± 3.88 | 9.33 ± 3.88 | 9.32 ± 3.88 | 9.33 ± 3.88 |
|  | Median (Q1, Q3) | 10.44 (5.40, 12.23) | 10.46 (5.40, 12.20) | 10.46 (5.39, 3.88) | 10.44 (5.40, 12.22) | 10.45 (5.40, 12.21) |
| MAX kinematic error (cm) | Mean ± SD | 0.006 ± 0.003 | 0.006 ± 0.003 | 0.006 ± 0.003 | 0.006 ± 0.003 | 0.006 ± 0.003 |
|  | Median (Q1, Q3) | .006 (.004, .008) | 0.006 (0.004, 0.007) | .006 (.004, .008) | 0.006 (0.004, 0.007) | 0.006 (0.004, 0.007) |
| MAX kinematic error (deg.) | Mean ± SD | 0.15 ± 0.25 | 0.14 ± 0.25 | 0.14 ± 0.25 | 0.17 ± 0.30 | 0.15 ± 0.25 |
|  | Median (Q1, Q3) | 0.06 (0.03, 0.08) | 0.06 (0.03, 0.08) | 0.06 (0.03, 0.09) | 0.06 (0.04, 0.10) | 0.06 (0.03, 0.08) |
| RMS kinematic error (cm) | Mean ± SD | 0.003 ± 0.001 | 0.003 ± 0.001 | 0.003 ± 0.001 | 0.003 ± 0.001 | 0.003 ± 0.001) |
|  | Median (Q1, Q3) | 0.003 (0.002, 0.004) | 0.003 (0.002, 0.004) | 0.003 (0.002, 0.004) | 0.003 (0.002, 0.004) | 0.003 (0.002, 0.004) |
| RMS kinematic error (deg.) | Mean ± SD | 0.07 ± 0.11 | 0.07 ± 0.12 | 0.07 ± 0.11 | 0.07 ± 0.11 | 0.07 ± 0.11 |
|  | Median (Q1, Q3) | 0.03 (0.02, 0.04) | 0.03 (0.02, 0.04) | 0.028 (0.016, 0.043) | 0.03 (0.02, 0.04) | 0.03 (0.02, 0.04) |
| MAX Joint Reserve (Nm) | Mean ± SD | 3.32 ± 8.28 | 3.31 ± 8.28 | 3.29 ± 8.22 | 3.30 ± 8.26 | 3.31 ± 8.26 |
|  | Median (Q1, Q3) | 0.08 (0.04, 2.06) | 0.07 (0.04, 2.06) | 0.07 (0.04, 2.10) | 0.07 (0.04, 2.12) | 0.07 (0.04, 2.16) |
| RMS Joint Reserve (Nm) | Mean ± SD | 0.57 ± 1.61 | 0.57 ± 1.61 | 0.57 ± 1.61 | 0.57 ± 1.61 | 0.57 ± 1.61 |
|  | Median (Q1, Q3) | 0.03 (0.02, 0.21) | 0.03 (0.02, 0.23) | 0.03 (0.02, 0.21) | 0.03 (0.02, 0.22) | 0.02 (0.02, 0.23) |
| Peak-Joint-Reserve-to-Peak-Joint-Torque Ratio (%) | Mean ± SD | 4.36 ± 8.59 | 4.36 ± 8.59 | 4.34 ± 8.56 | 4.35 ± 8.58 | 4.36 ± 8.58 |
|  | Median (Q1, Q3) | 0.15 (0.06, 4.15) | 0.14 (0.06, 4.16) | 0.13 (0.06, 4.16) | 0.14 (0.06, 4.16) | 0.13 (0.06, 4.17) |

The following figures show comparison of lower extremity electromyography (EMG) signals to simulated activation resulting from computed muscle control (CMC). EMG signals were normalized to maximum values obtained during maximum voluntary isometric contraction testing to best represent the 0 (none) – 1 (full activation) scale used in CMC. The timing of peaks and general shapes of the curves should align between EMG signals and CMC activation, but it is not expected that magnitudes will align [2].

Figure S2.1: Comparison of CMC activation to EMG signal normalized to maximum values obtained during maximum voluntary isometric contraction testing for representative participant “A”.

Figure S2.2: Comparison of CMC activation to EMG signal normalized to maximum values obtained during maximum voluntary isometric contraction testing for representative participant “B”. Vastus medialis was not included due to poor EMG quality upon inspection of the raw signal.

Figure S2.3: Comparison of CMC activation to EMG signal normalized to maximum values obtained during maximum voluntary isometric contraction testing for representative participant “C”. Rectus femoris was not included due to poor EMG quality upon inspection of the raw signal. S2

References

[1] Hicks J. Simulation with OpenSim - Best Practices. OpenSim Documentation. 2012. Available: <https://opensimconfluence.atlassian.net/wiki/spaces/OpenSim/pages/53087686/Simulation+with+OpenSim+-+Best+Practices.>

[2] Hicks, J. L., Uchida, T. K., Seth, A., Rajagopal, A., & Delp, S. L. (2015). Is My Model Good Enough? Best Practices for Verification and Validation of Musculoskeletal Models and Simulations of Movement. Journal of Biomechanical Engineering, 137(2), 209051. 10.1115/1.4029304
