# Supplementary material for: Effect of altered gluteus maximus strength on the magnitude and direction of hip joint contact forces during simulations of gait
Source: PLoS One. 2025 Jun 23;20(6):e0324451. doi: 10.1371/journal.pone.0324451 (PMC12184943; doi:10.1371/journal.pone.0324451)

S3 Appendix: Muscle force production from hip extensors under each gluteus maximus strength condition

The following images show the group average for force production (normalized to body weight) under each gluteus maximus strength condition for all extensor muscles as defined in the Gait2392 model.


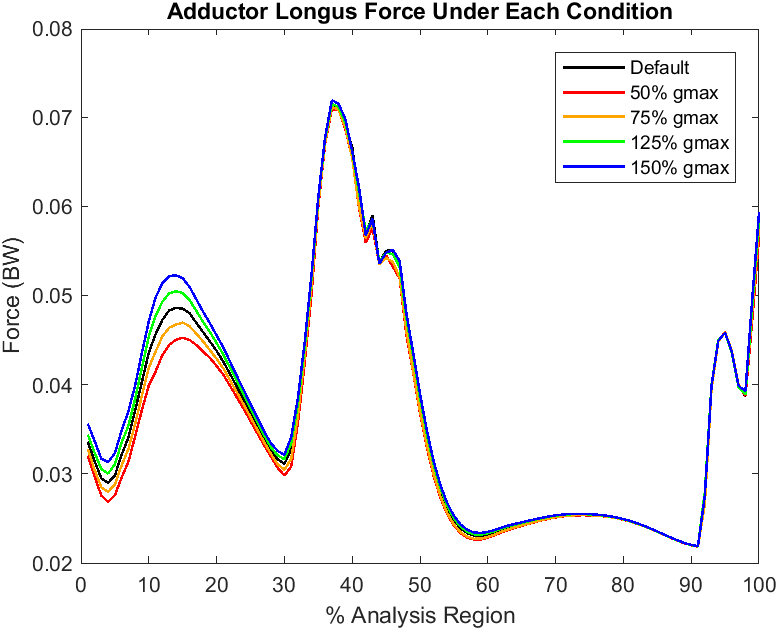

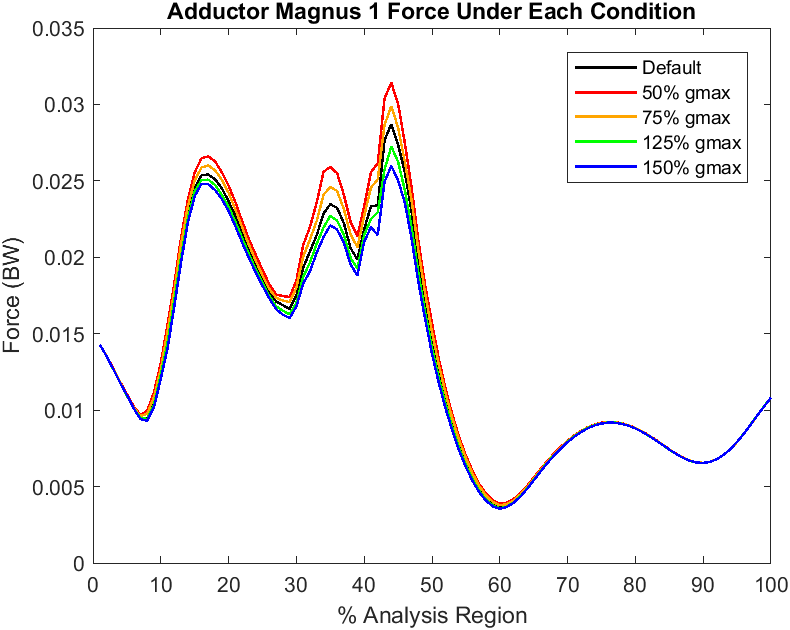


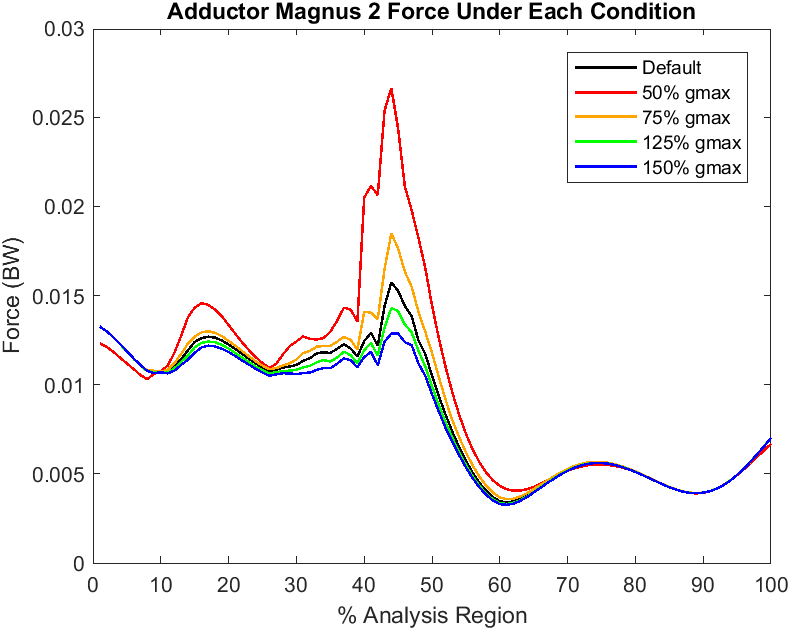

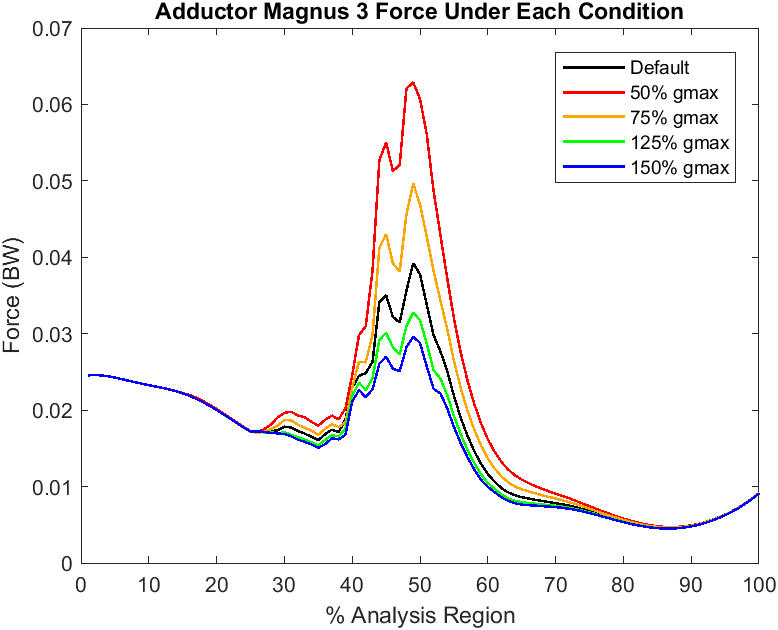


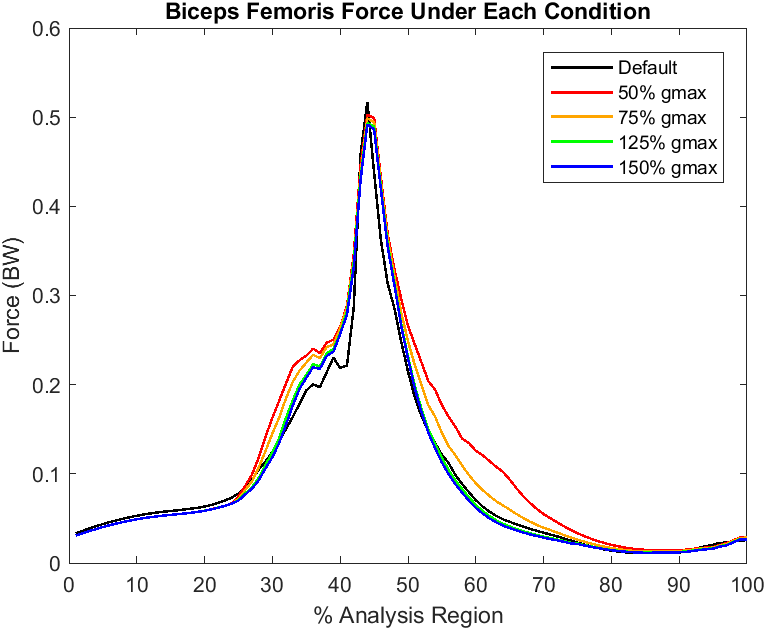

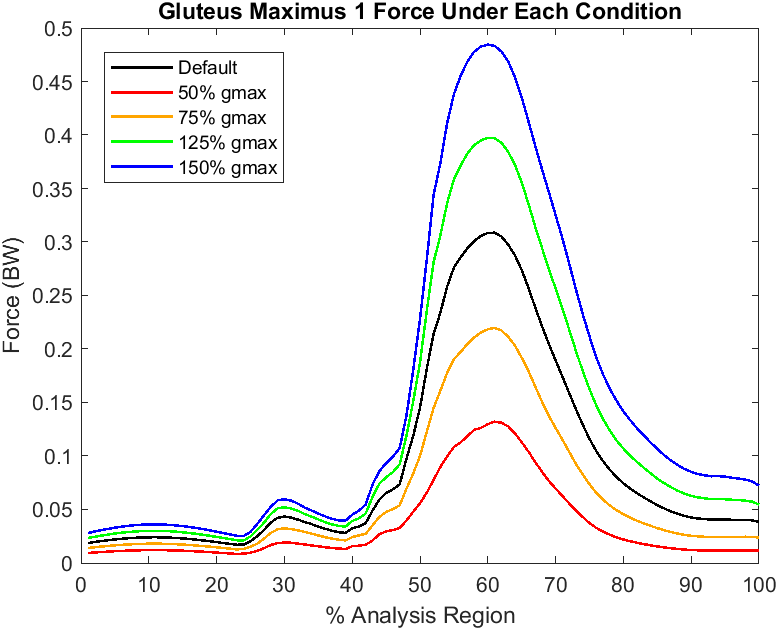


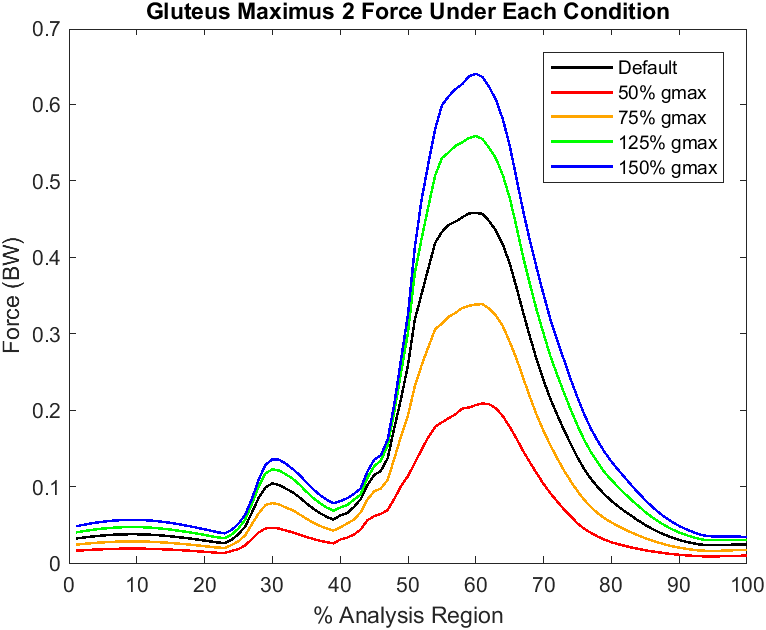

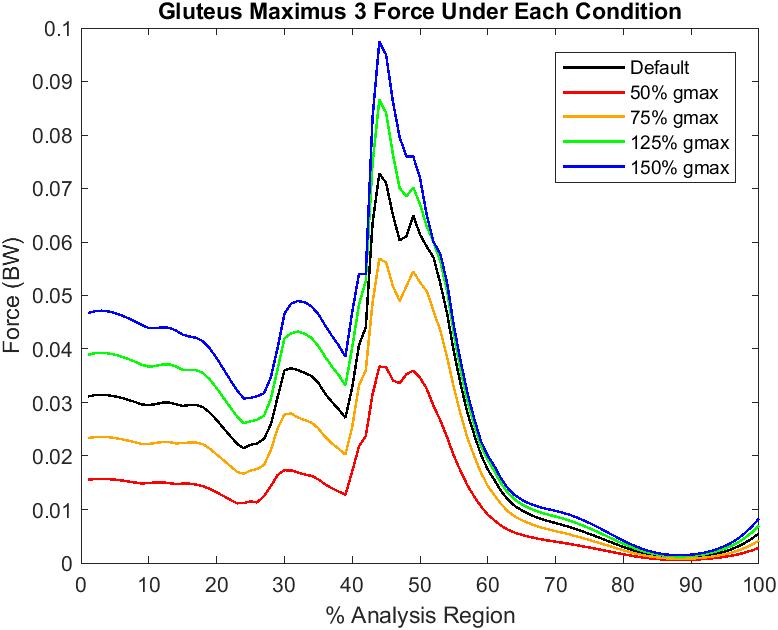


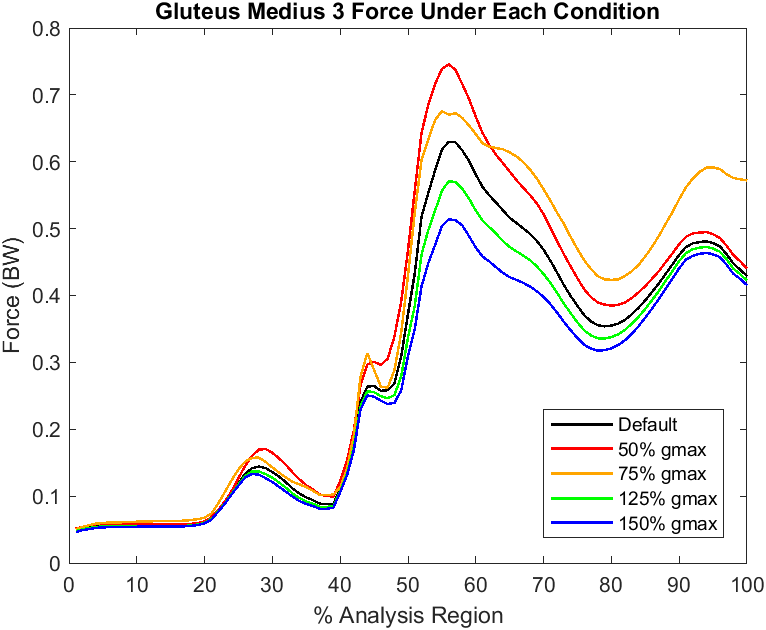

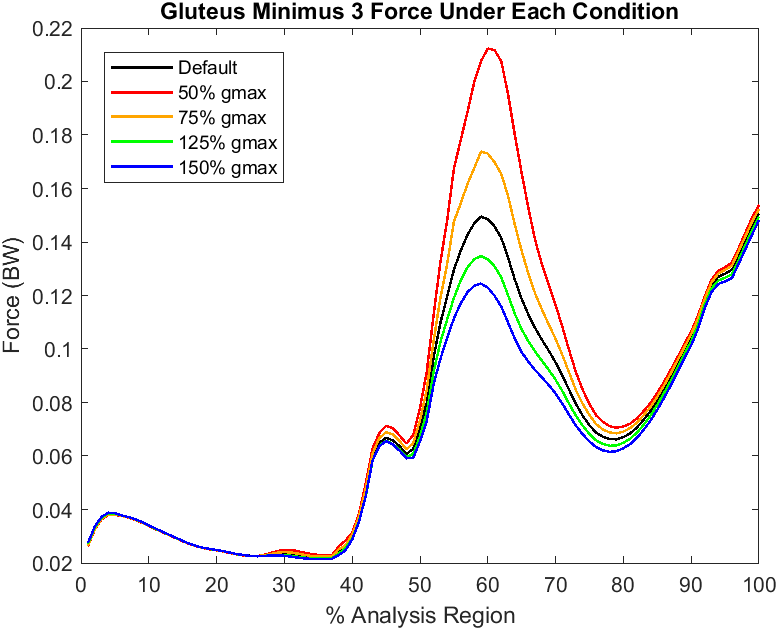


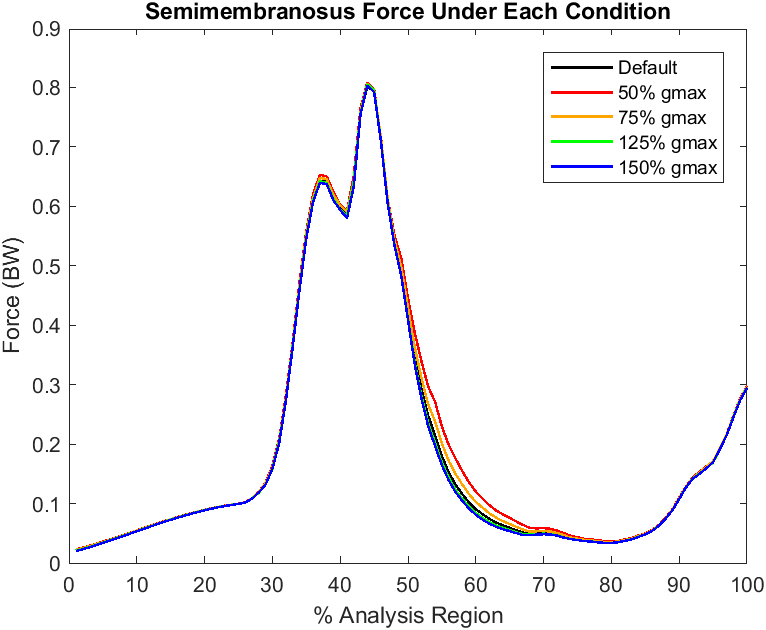

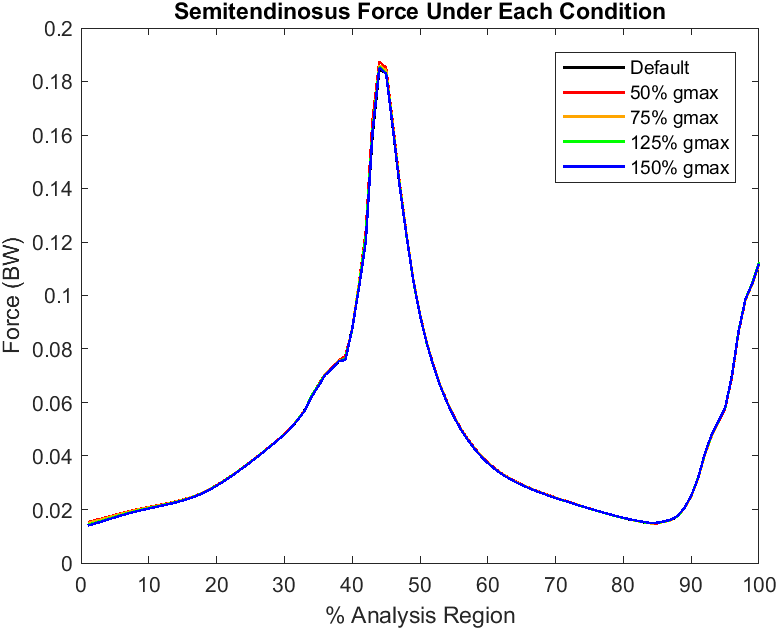

Supplement: S3 Appendix — (DOCX) [file pone.0324451.s003.docx]
