# Supplementary material for: Effect of altered gluteus maximus strength on the magnitude and direction of hip joint contact forces during simulations of gait
Source: PLoS One. 2025 Jun 23;20(6):e0324451. doi: 10.1371/journal.pone.0324451 (PMC12184943; doi:10.1371/journal.pone.0324451)
Supplement: S4 Appendix — (DOCX) [file pone.0324451.s004.docx]

S4 Appendix – Changes in Activations and Forces


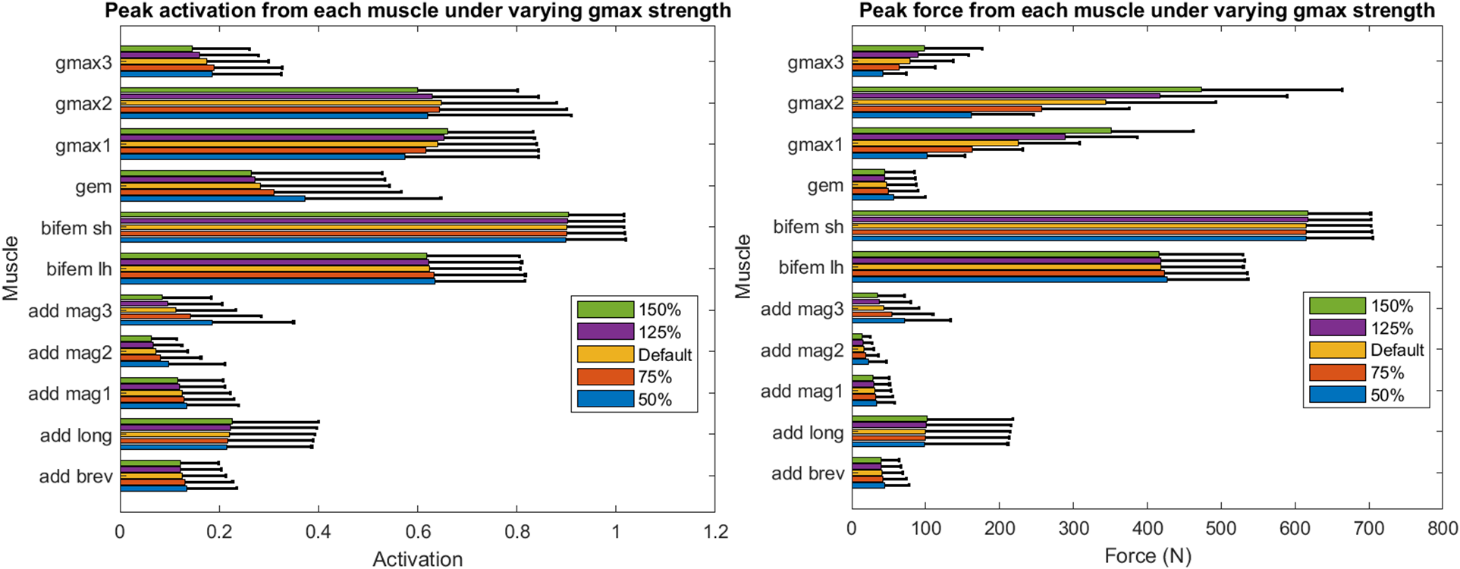

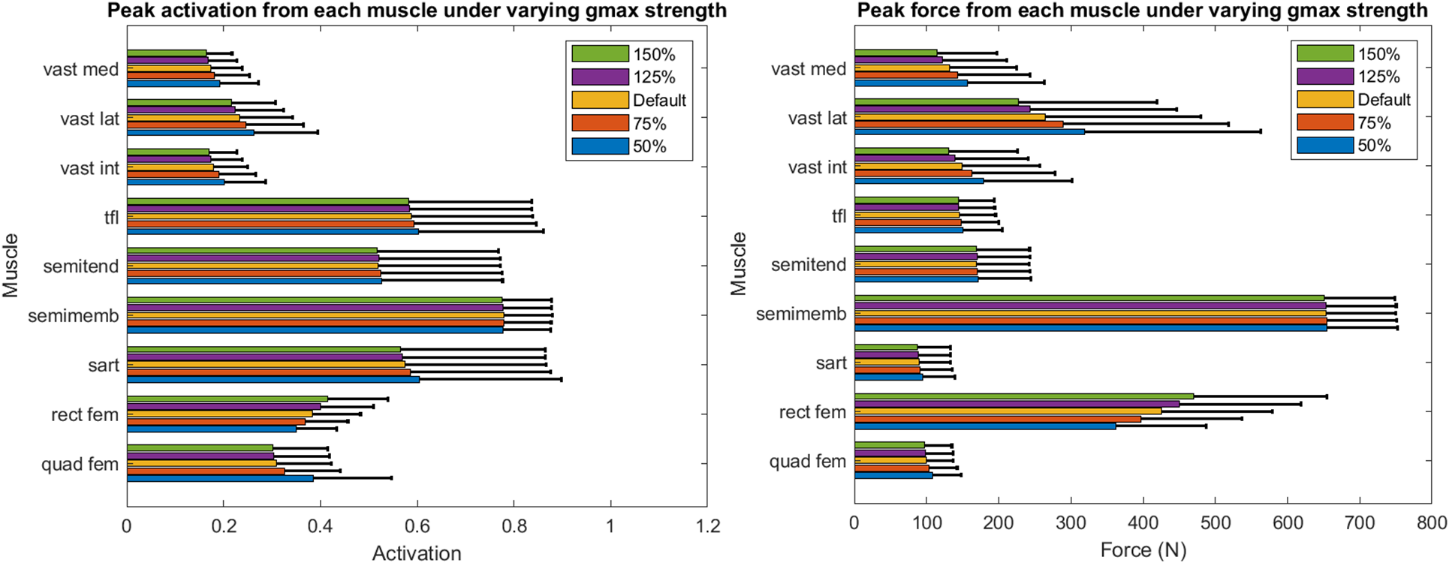

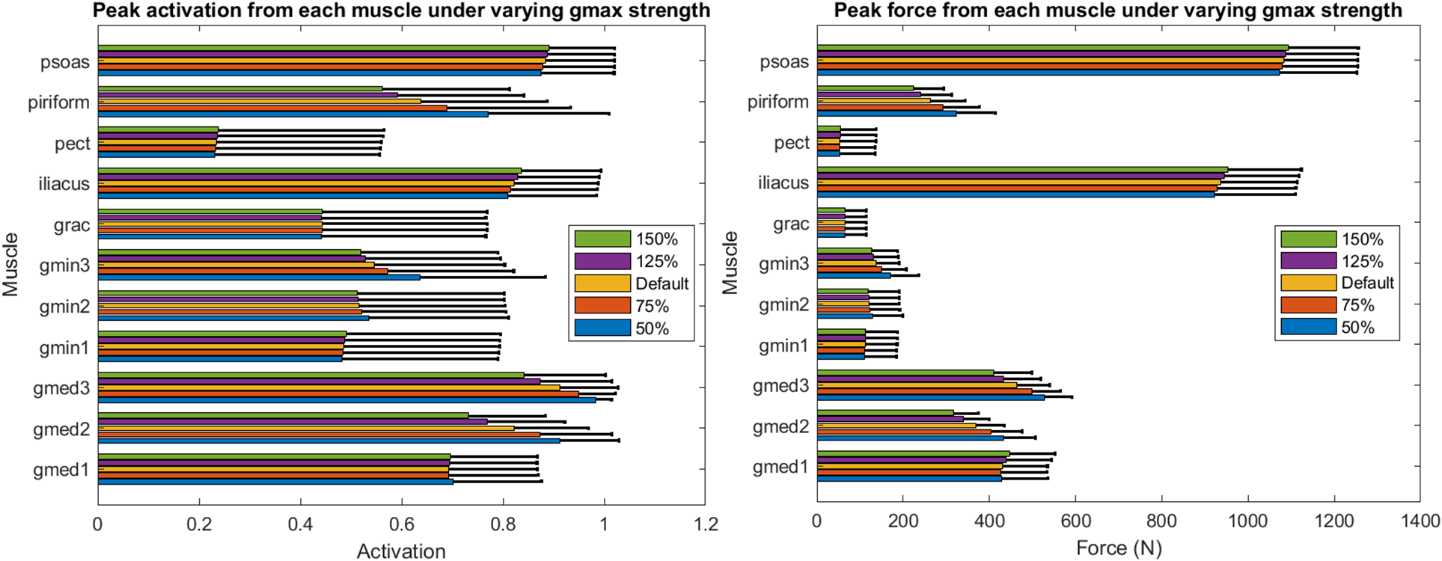


Figure S4.1: Peak activation (0- no activation, 1-full activation) and peak force (N) from all hip and thigh muscles under each condition of gluteus maximus strength.


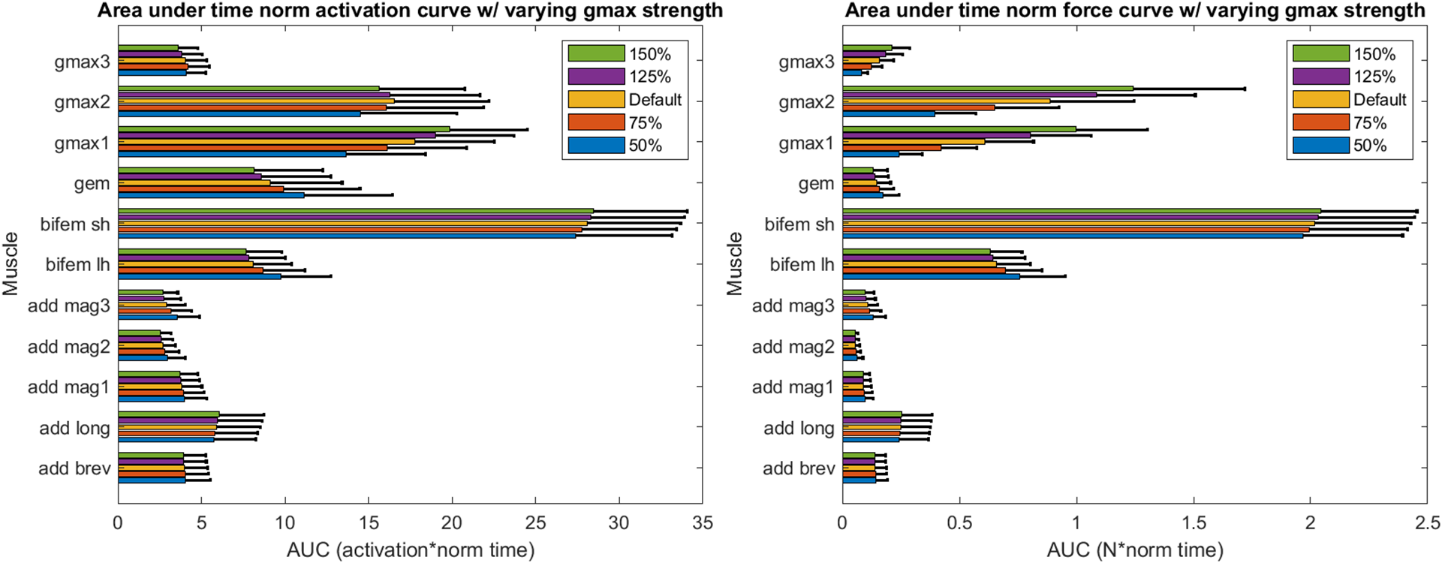

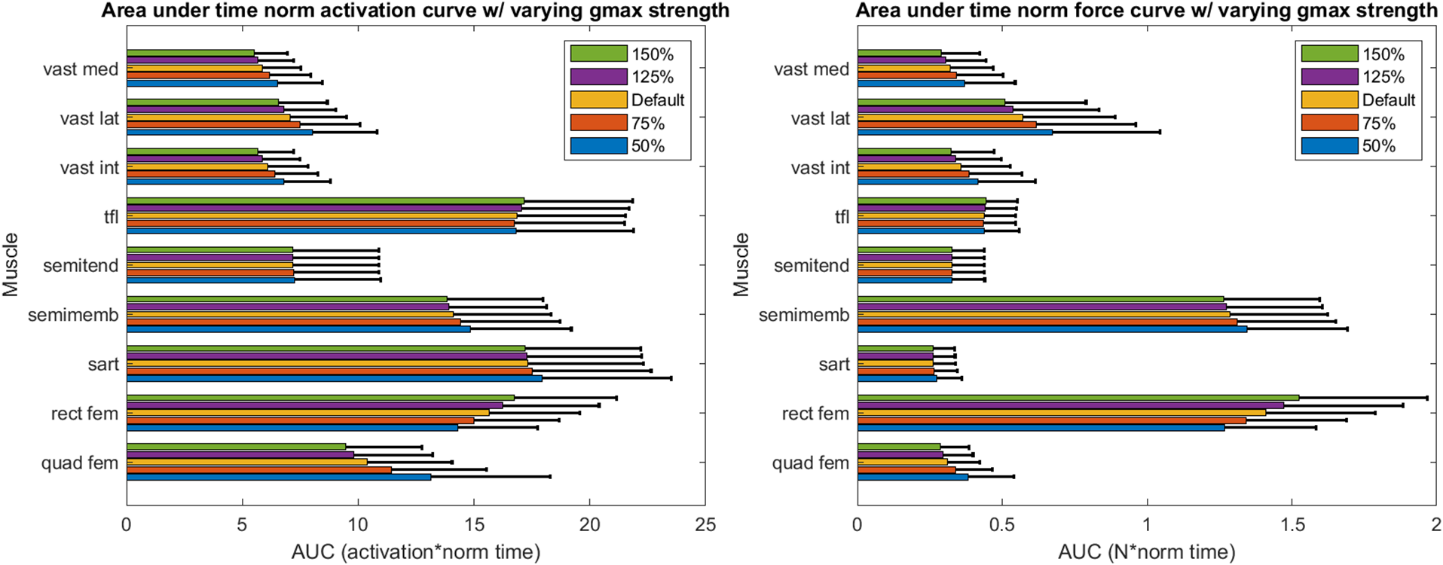

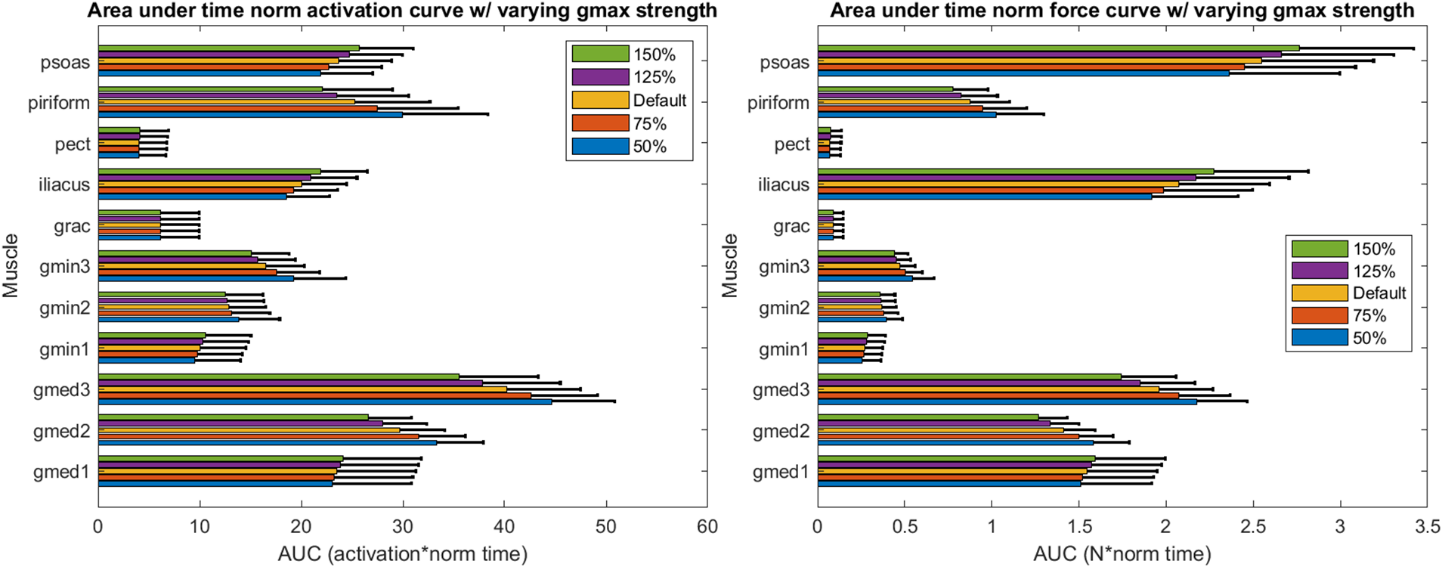


x10^4^

x10^4^

x10^4^

Figure S4.2: Area under the time normalized activation (0- no activation, 1-full activation) and force (N) curves for all hip and thigh muscles under each condition of gluteus maximus strength. Scale for area under force curve values is x10^4^.
