# Supplementary material for: Effect of altered gluteus maximus strength on the magnitude and direction of hip joint contact forces during simulations of gait
Source: PLoS One. 2025 Jun 23;20(6):e0324451. doi: 10.1371/journal.pone.0324451 (PMC12184943; doi:10.1371/journal.pone.0324451)
Supplement: S5 Appendix — (DOCX) [file pone.0324451.s005.docx]

S5 Appendix: Figures displaying results for group comparisons (FAIS vs HC) and complete timeseries results for statistical nonparametric mapping


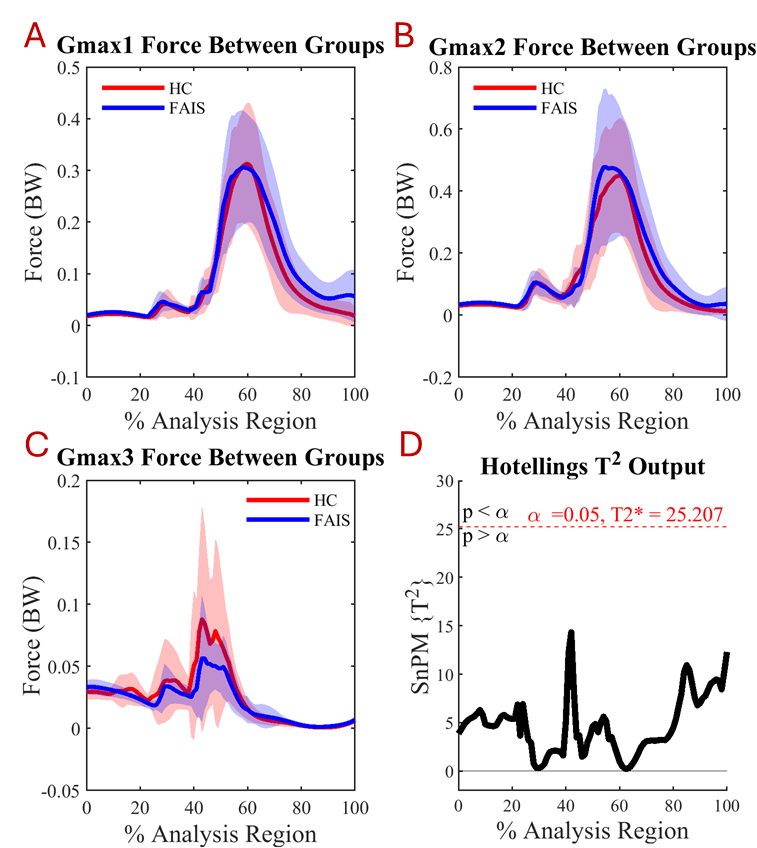


Figure S5.1: A-C: Simulated gluteus maximus, superior (gmax1), middle (gmax2), and most inferior (gmax3), force comparison between FAIS and healthy control (HC) groups. D: SnPM Hotelling’s T^2^ trajectory. The horizontal line representing the critical field theory threshold of T^2^ = 25.207.


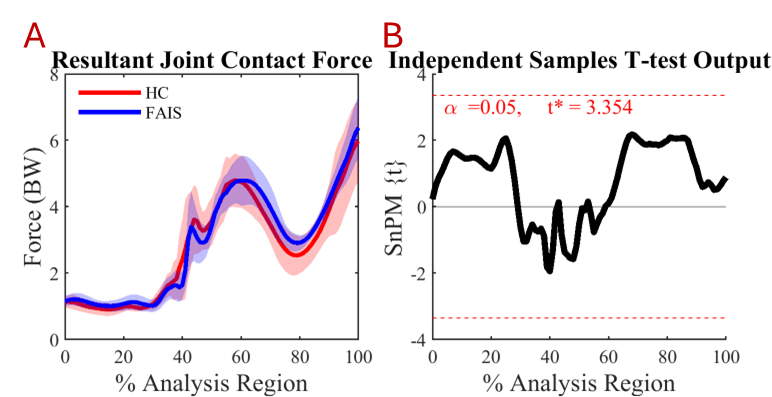


Figure S5.2: Resultant JCFs compared between FAIS and healthy control (HC) groups (A) and SnPM t-test performed for resultant JCF vector comparison with a t* value = 3.354 (B).


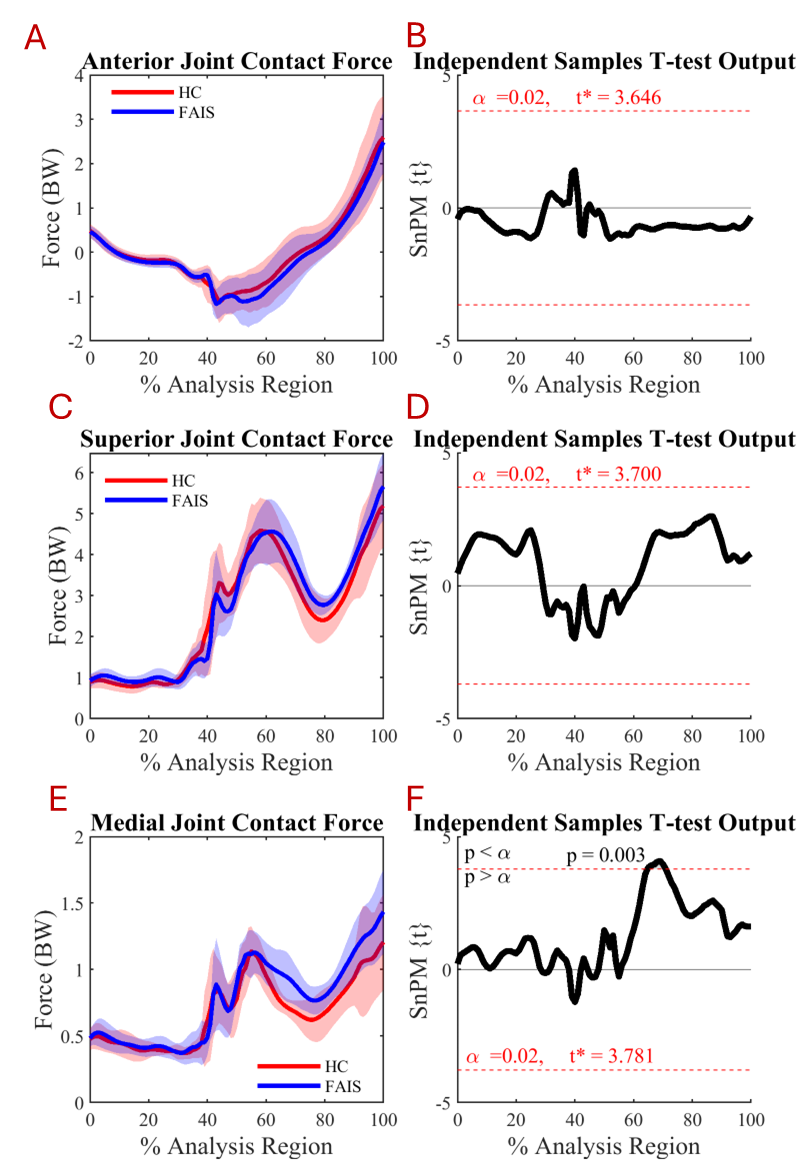


Figure S5.3: Anterior (A), superior (C), and medial (E) components of JCFs compared between individuals with FAIS and healthy controls (HC) (left) and their corresponding SnPM t-test (B, D, F) (right). Individuals with FAIS demonstrated larger medial JCFs than healthy controls from 65-71% of the analysis region (p = .003).


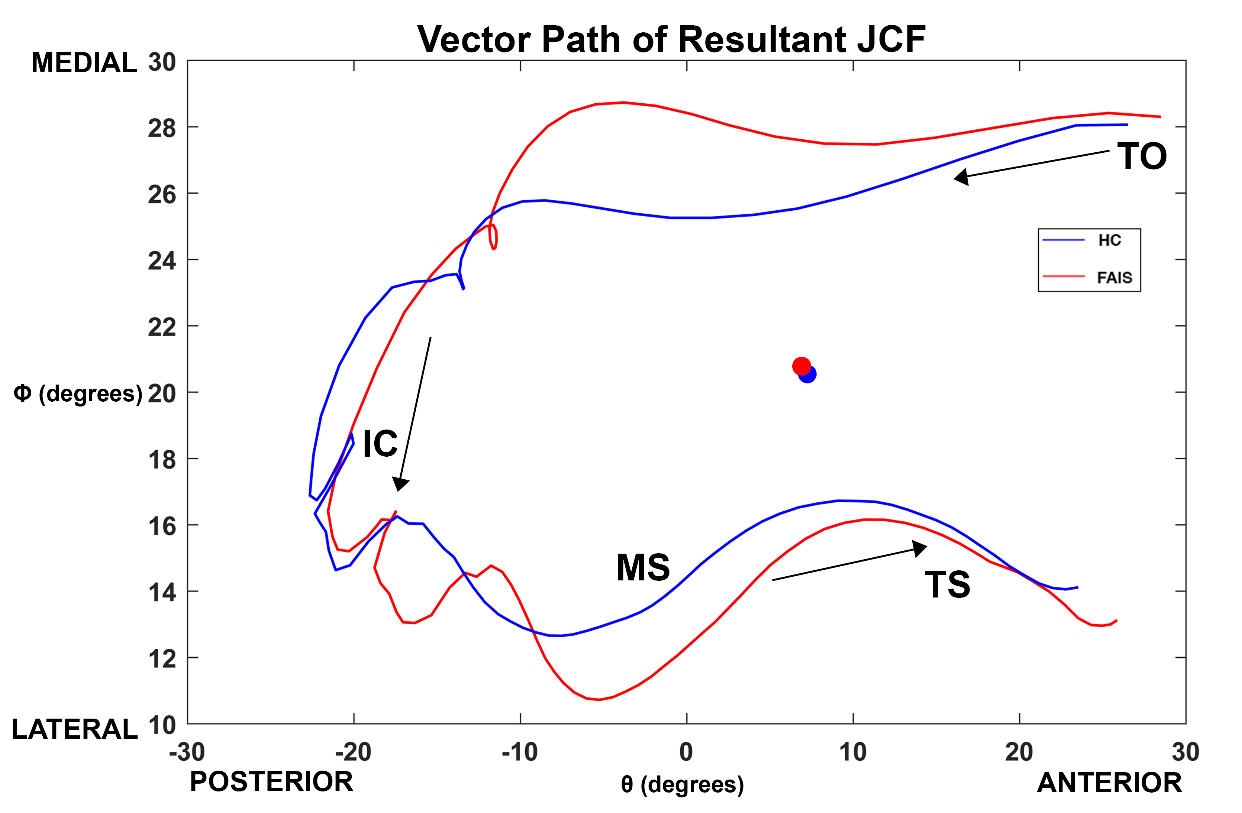


Figure S5.4: Theta (Θ) vs Phi (Φ) joint contact force (JCF) vector path (angle-angle plot) of individuals with FAIS compared to healthy controls (HC). Gait cycle labels are placed for orientation at beginning and end of vector path: TO = involved toe off (start of analysis region), IC = initial contact, MS = midstance, TS = terminal stance (end of analysis region). Color-coordinated circles represent regional loading centers for each group calculated per least squares algorithm. Regional loading centers did not differ between the FAIS and healthy control groups (p ≥ .812).


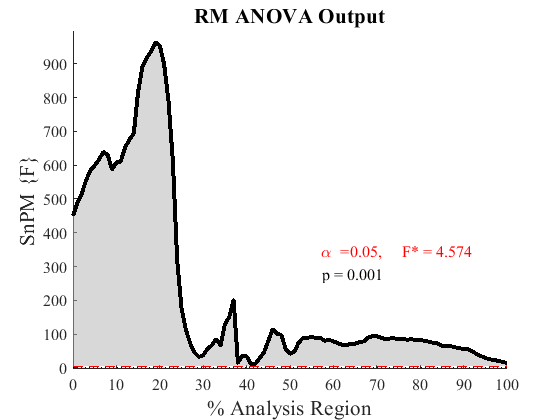


Figure S5.5: ANOVA results for the magnitude of the resultant JCF under the 5 different strengthening conditions. The shaded region below the SnPM{*F*} trajectory and above the random field theory computed critical *F* threshold for significance of *F** = 4.574 shows the significant suprathreshold cluster from 0-100% of the analysis region (p = 0.001).


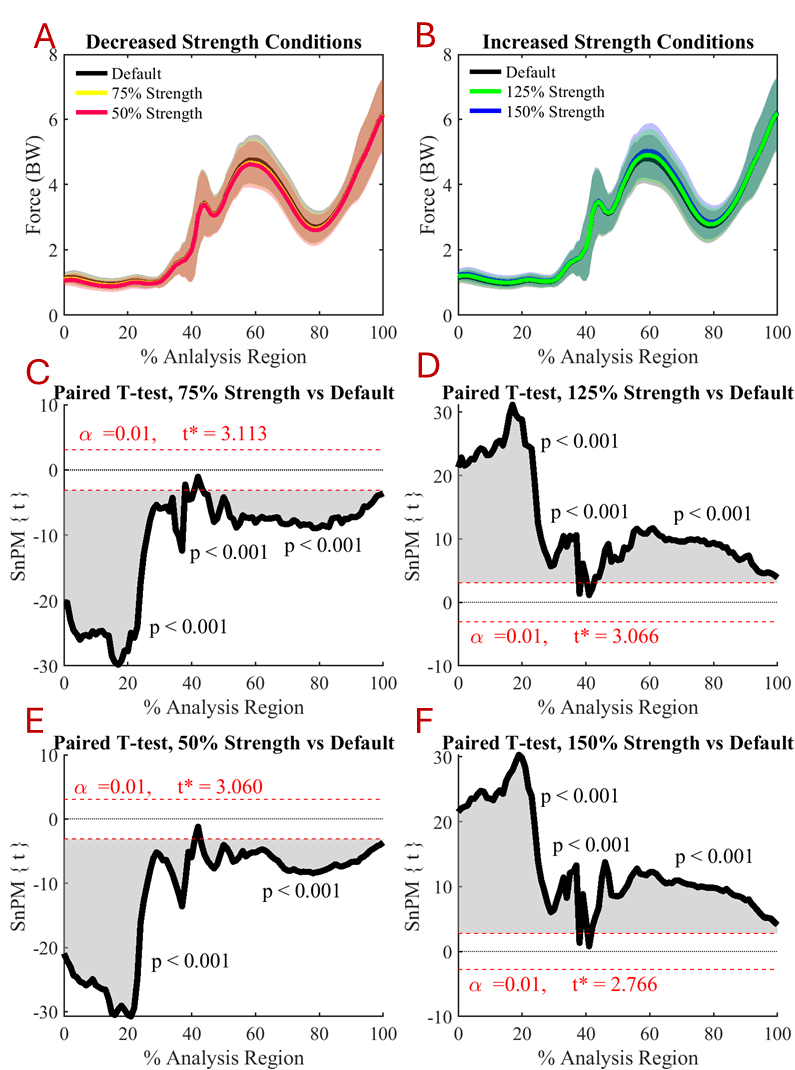


Figure S5.6: A: Default and weakened condition groups (75% & 50%) resultant joint contact force output normalized to BW. B: Default and strengthened condition groups (125% & 150%) resultant joint contact force output normalized to BW. C&E: Post hoc SnPM paired samples t-tests between 75% and 50% compared to default condition, t* values = 3.066, 3.122 respectively. E&F: Performed post hoc SnPM paired samples t-tests 75% and 50% compared to default condition, t* values = 2.902, 2.915 respectively.


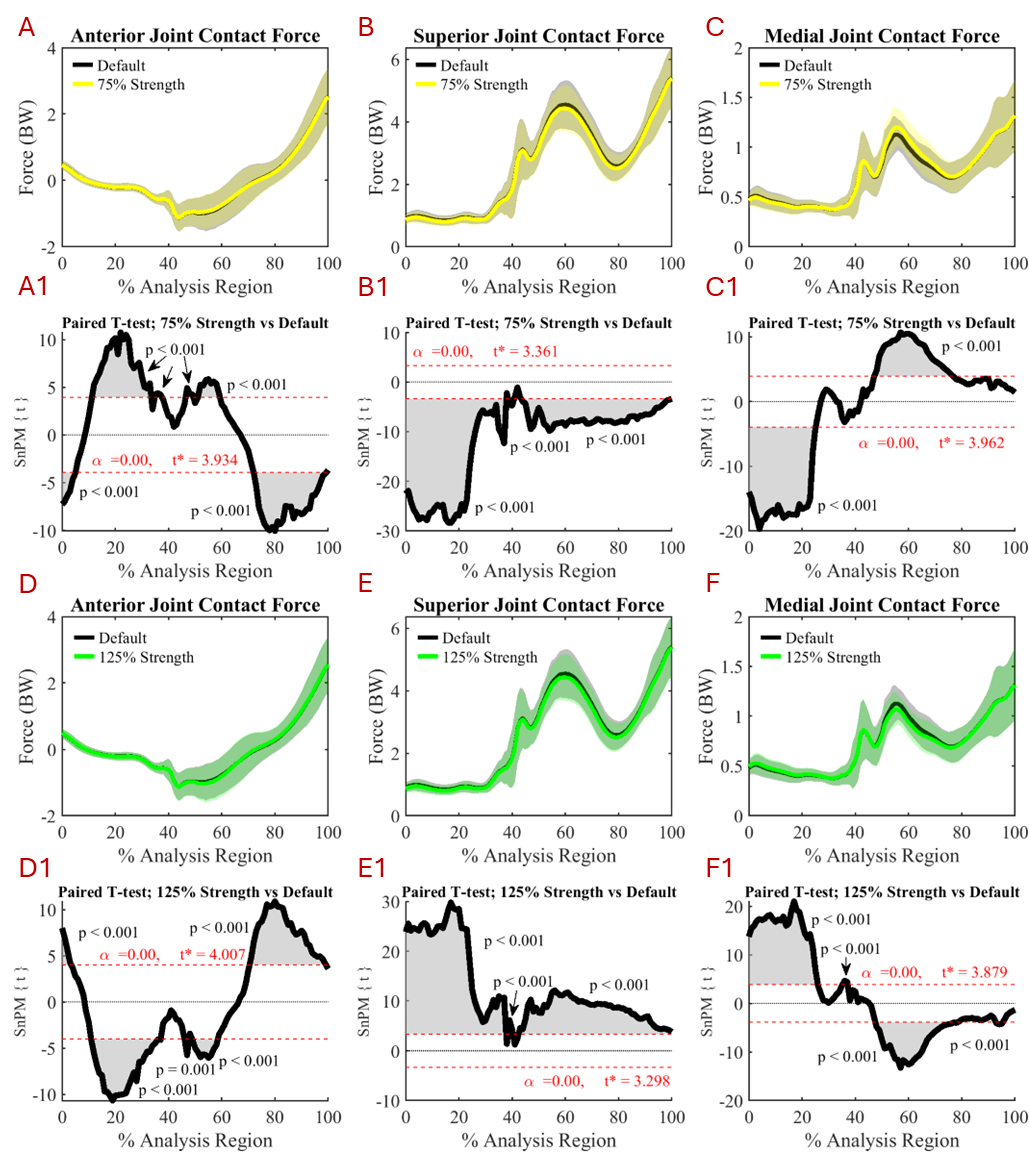


Figure S5.6: A-C: Anterior, superior, and medial components of JCFs normalized to BW for the 75% condition compared to default. A-C1: Corresponding SnPM paired samples t-tests per each component. Horizontal dashed lines representing the critical random field theory thresholds of t* = 3.934, 3.361, 3.962 for anterior, superior, and medial comparisons, respectively of the 75% condition respectively. (+) shaded portions representing areas of significant increases in JCFs and (-) representing areas of significant decreases in JCFs in comparison to default. D-F: Anterior, superior, and medial components of JCFs normalized to BW for the 125% condition compared to default. D-F1: Corresponding SnPM paired samples t-tests performed per each component. Horizontal dashed lines representing the critical random field theory thresholds of t* = 4.007, 3.298, 3.879 for anterior, superior, and medial component comparisons, respectively in the 125% condition to default. With a Bonferroni correction for multiple comparisons (12 total), α = 0.0043.
