# Supplementary material for: Effect of altered gluteus maximus strength on the magnitude and direction of hip joint contact forces during simulations of gait
Source: PLoS One. 2025 Jun 23;20(6):e0324451. doi: 10.1371/journal.pone.0324451 (PMC12184943; doi:10.1371/journal.pone.0324451)
Supplement: S11 Files — (ZIP) [file pone.0324451.s011.zip › V3D_Osim_Files/Instructions for Running Visual3D to OpenSim_4_0.docx]

Visual3D

c-motion | Support@c-motion.com

OpenSim 4.0

Release notes for OpenSim

The revised “**Instructions for Running Visual3D to OpenSim Integration**” section of this document gives the exact details on running the new OpenSim RRA tool and the CMC Tools.

Changes for exporting from Visual3D to OpenSim version 4.0 and newer:

1. When running OpenSim 4.0, you must load the GRF mot file using the steps described here

**­Table of Contents:**

1. [Exporting Data from Visual3D to OpenSim](#Exporting)
   1. [Preface](#Preface)
   2. [Introduction](#Introduction)
2. [Modeling Requirements](#Modeling)
3. [Visual3D Scaling](#Scaling)
4. [Instruction for Running Visual3D to OpenSim Export](#ExportInstructions)
5. [Appendix A: v3d_lab](#AppendixA_v3dlab)
6. [Appendix B: Visual3D and OpenSim xml Files Modifications](#AppendixB_Files)

**Exporting Data from Visual3D to OpenSim**

**Preface**

Visual3D allows the user to export an OpenSim compatible .mot file, which can be used as input to OpenSim gait simulations.

The Visual3D to OpenSim integration is based on the OpenSim tutorial “*OpenSim_GeneratingMuscleActuatedSimulation.pdf*”. **It is assumed that the user is familiar with this tutorial and the files used in this process.**

This PDF is provided in as part of the Visual3DtoOpenSim_4_0 download file.

The default OpenSim model used in the Visual3D to OpenSim process is the model gait2392_simbody.osim, which is distributed with the OpenSim software, and part of this download package.

**Introduction**

Visual3D 5.0 allows the user to export OpenSim compatible motion files designed for use with OpenSim gait models. To accomplish this Visual3D creates an OpenSim compatible motion file, Visual3d_SIMM_input.mot, which bypasses the Scaling and Inverse Kinematics (IK) in OpenSim and can be used directly by the OpenSim Residual Reduction Algorithm.

Visual3D’s ability to write OpenSim compatible .mot files is based on Visual3D’s own IK algorithm. The theory behind the Visual3D to OpenSim export is a three-stage process:

1. Visual3D computes the scale factors, which scale the OpenSim gait model to the Visual3D static calibration model. For all OpenSim versions, these scale factors get written to a special OpenSim scale set file named Visual3d_Scale_ScaleSet.xml. (See the [Visual3D Scaling](#Scaling) section of this document for more details.)
   1. For **OpenSim v3.1**, the user must run the Visual3D pipeline OpenSim_Scale_Factors.v3s. Download the Visual3DtoOpenSimv3.2.0 package for this pipeline and more details.
2. Visual3D uses IK to fit the scaled OpenSim gait model to the Visual3D Static Calibration Model. (This is similar to the OpenSim Marker Placer Process).
3. Visual3D then uses IK to fit the gait data to the newly calibrated model.

**Modeling** **Requirements**

**The Following model requirements must all be met. Please read the three requirements carefully:**

1. The Visual3D model used for OpenSim output **must include the following segments:**

| right foot (RFT) | left foot (LFT) |
| --- | --- |
| right shank (RSK) | left shank (LSK) |
| right thigh (RTH) | left thigh (LTH) |
| pelvis (RPV) |  |
| Thorax/Ab (RTA) |  |

1. T**he proximal end of the Thorax/Ab (RTA) must be created at the exact same location as the proximal end of the pelvis**.
   1. The proximal end of the RTA should be at the same location as the proximal end of the pelvis, and the distal end of the RTA should be approximately at the height of the shoulders.
   2. The Z-axis (which goes distal to proximal) will now be pointed downward and the Y-axis pointed backward. The orientation must be changed so it matches the local coordinate system of the other segments. To do this you:
      1. Click “Modify the Segment Coordinate System” in the “Segment Properties” tab.
      2. Set the “AP Axis” to –Y and the “Distal to Proximal” to –Z.
2. The subject must either be walking in the +Y direction of the lab (+Z vertical) OR a virtual lab called v3d_lab must be created.
   1. See [Appendix for example of v3d_lab](#AppendixA_v3dlab)

**Visual3D Scaling**

For all OpenSim versions (**EXCEPT v3.1**) Visual3D automatically scales the OpenSim model by:

1. Changing the OpenSim scaling method to **manual scaling** for all segments. (The simplified version of the OpenSim scaling setup file, called Visual3d_Setup_Scale.xml, should be used to scale the model in OpenSim.)
2. Updating the file Visual3D_Scale_ScaleSet.xml to include the Visual3D computed scale factors for the pelvis, left and right thigh and left and right tibia segments.

For OpenSim **version 3.1 ONLY**, Visual3D scales the OpenSim model by:

1. The user must run the Visual3D pipeline OpenSim_Scale_Factors.v3s which writes the scale factors to a file called OpenSim_Scaling.txt
2. In OpenSim the user must change all of the scaling factors to manual and then enter the scale factors in OpenSim_Scaling.txt by hand. It is recommended that the average of the left and right thigh scale factors (TH_MEAN in OpenSim_Scaling.txt) be used to scale the torso, caln_r, calcn_l, toes_r and toes_l segments in OpenSim.

**Important Note: The manual scale factors written to the Visual3d_Scale_ScaleSet.xml file must be used to scale the pelvis, thigh and tibia segments in OpenSim. If these scale factors are not used then center of pressure, which is also written by Visual3D to the Visual3d_SIMM_input.mot, will not be adjusted properly in relation to the model. (The scale factors for the feet and torso do not affect the relationship between the foot and the center of pressure.)**

The manual scale factors computed by Visual3D are based on the subject calibration data and the OpenSim segment geometry (as specified in the gait2392_simbody.osim). Specifically, the segments are scaled by:

- **Pelvis:** To scale the pelvis Visual3D computes the distance (in meters) between the hip centers in the subject calibration and divides this value by 0.167. The value 0.167 is the distance between the medial and lateral hip location in the file gait2392_simbody.osim.
  - Note: The default OpenSim scales the pelvis based on the distance between the left and right ASIS; however since Visual3D does not require targets be placed on the left and right ASIS the pelvis is scaled based on the distance between the hip joint centers which will always be present.
- **Thigh:** To scale the thigh Visual3D computes the distance between the hip and knee joint center in the subject calibration and divides this value by 0.396. The value 0.396 is the distance between the hip and knee location in the file gait2392_simbody.osim.
  - Note: the knee location will vary with knee joint angle in OpenSim and the value 0.396 represents the knee location at zero degree of knee flexion.
- **Shank:** To scale the thigh Visual3D computes the distance between the knee and ankle joint center in the subject calibration and divides this value by 0.43. The value 0.43 is the distance between the knee and ankle location in the file gait2392_simbody.osim.
- **Torso, Calcaneus and Toes:** Because most users build their feet without markers at the very end of the foot and at the top of the head and thus using the average thigh length is used to scale these segments.
  - Physical anthropometry studies have shown thigh length is somewhat strongly correlated with height.

**Instruction for Running Visual3D to OpenSim Export**

**Part 1 – Creating the motion file for OpenSim.**

1. Start Visual3D and create a Visual3D hybrid model containing the following segments:

| right foot (RFT) | left foot (LFT) |
| --- | --- |
| right shank (RSK) | left shank (LSK) |
| right thigh (RTH) | left thigh (LTH) |
| pelvis (RPV) |  |
| Thorax/Ab (RTA) |  |

1. If the subject is **not walking in the +Y** with +Z as vertical **create a v3d_lab** segment as outlined in the [Appendix](#AppendixA_v3dlab)
2. It is highly recommended that only one C3D file be loaded in your CMZ file before exporting. The C3D file you’re exporting should be the only file in the workspace.
3. Go to **File** and select **Export🡪Export OpenSim Motion File.** Alternatively, the user could export the file using **Export🡪Export OpenSim Motion File without Cleanup.** (The only difference between the two methods is that if the method without Cleanup is selected then the OpenSim Inverse Kinematics remains applied to the model after export and the resulting position state variables get written to the “DERIVED🡪Inverse Kinematics” folder of Visual3D “Signal and Events” data tree.)
4. Enter the data range and click the **OK** button.
5. Visual3D now needs the required **Visual3d_Scale_ScaleSet.xml** file. If this xml is in the same folder as the CMZ, it will be automatically loaded. If it’s not, a File Selection Dialog will appear to select the file.
6. Then Visual3D applies the Inverse Kinematics process to the gait data. (This can take several minutes.)

Once steps 1-7 are completed, Visual3D will have:

- Updated the **Visual3d_Scale_ScaleSet.xml** file
- Written the **Visual3d_SIMM_input.mot** file
- Written the **Visual3d_SIMM_grf.mot** file

**Part 2 – Scaling and Viewing the motion file for OpenSim**

1. Start OpenSim and load the file OpenSim model (**gait2392_simbody.osim**).
2. From the Tools pull down select “Scale Model”
3. In the Scale Tool Dialog select Settings -> Load Settings
   1. Open the file **Visual3d_Setup_Scale.xml**
   2. Uncheck the Adjust Model Markers box
   3. Hit the Run button to create the scaled model.
   4. Hit the close button to close the dialog.
   5. The model will be called Visual3D in the Navigator window.
4. Right click on model 3DGaitModel2354 in the navigator window and select Close.
   1. A dialog will appear – select “No” so you do not save settings.
   2. Vusial3D will now be the only Model
5. From the File pull-down select Load Motion and load Visual3D_SIMM_input.mot
6. [If using OpenSim 4.0, in the Navigator pane, expand Visual3d, expand Motions, right click on Visual3D_SIMM_input.mot](https://simtk-confluence.stanford.edu:8443/display/OpenSim/Associating+Data+with+a+Motion?searchId=H31YOZ3BP#AssociatingDatawithaMotion-AssociateMotionData)
   1. Select Associate Motion Data
   2. Select Visual3D_SIMM_grf.mot
7. Hit the play button to Animate (On my computer I set the speed to 0.2)

**Part 3 – Running the Residual Reduction**

1. From the Tools pull down select “Reduce Residuals”
2. In the RRA Tool Dialog select Settings -> Load Settings
3. Open the file Visual3D_Setup_RRA.xml
4. Adjust the “Time range to Process” to the desired start and end time
5. Select the Actuators and External Loads Tab
6. Click RUN to start RRA
7. Click Close to close the RRA Dialog Tool

**Part 4 – Running Computed Muscle Control**

1. From the Tools pull down select “Compute Muscle Control “
2. In the CMC Tool Dialog select Settings -> Load Settings
3. Open the file Visual3D_Setup_CMC.xml
4. Adjust the “Time range to Process” to the desired start and end time
5. Select the Actuators and External Loads Tab
6. Click RUN to start CMC
7. Click Close to close the CMC Tool Dialog

**Appendix A: v3d_lab**

For the OpenSim Export, it’s necessary that the subject walk in the +Y direction of the lab (in the dynamic trial) OR a virtual lab is built. **The virtual lab MUST be called v3d_lab.**

This distribution contains two examples of a virtual lab (v3d_lab.mdh and Dynamic_v3d_lab.mdh). Either MDH file can be appended to your current model template (Model -> Append Model Template to current model). You may need to modify the lab to work with your specific data:

- v3d_lab.mdh assumes the subject is walking along the -X direction of the lab.
- Dynamic_v3d_lab.mdh assumes the subject is walking along the X direction of the lab (either +/-).

The provided example v3d_lab is setup for the case where the subject is walking along the -X direction of the lab in the dynamic trial (and +Z points up):

!

! Segment v3d_lab

!

HYBRID_SEGMENT

! /CALIBRATION_FILE=

/TYPE=Visual_3D

/NAME=v3d_lab

/REFERENCE_OBJECT=Medial Distal+Lateral Distal+Medial Proximal+Lateral Proximal+Proximal Joint+Distal Joint+Additional Object+Additional Plane+Distal Radius+Proximal Radius

/REFERENCE_OBJECT_NAMES=v3d_lab_med_distal+v3d_lab_lat_distal+v3d_lab_med_prox+v3d_lab_lat_prox++++Medial+v3d_lab_Distal_Radius+v3d_lab_Proximal_Radius

/REFERENCE_OBJECT_TYPES=LANDMARK+LANDMARK+LANDMARK+LANDMARK+++++METRIC+METRIC

/USE_CAL_TARGETS_FOR_TRACKING=FALSE

/TRACKING_TYPES=LANDMARK+LANDMARK+LANDMARK+LANDMARK

/TRACKING_NAMES=v3d_lab_lat_prox+v3d_lab_med_prox+v3d_lab_lat_distal+v3d_lab_med_distal

! /USE_OPTIMAL_TRACKING=TRUE

/KINEMATIC_ONLY=TRUE

! /STATIC_MODEL=FALSE

/GRAPHICS_ROT_X=0

/GRAPHICS_ROT_Y=0

/GRAPHICS_ROT_Z=0

/GRAPHICS_SCALE_X=1

/GRAPHICS_SCALE_Y=1

/GRAPHICS_SCALE_Z=1

/GRAPHICS_TRANSLATE_X=0

/GRAPHICS_TRANSLATE_Y=0

/GRAPHICS_TRANSLATE_Z=0

! /GRAPHICS_UNIT_SCALE=1.000000

/MASS=0.000000

! /GEOMETRY=

/PROX_TO_CG_AXIAL=0.5*v3d_lab_SEG_LENGTH

/PROX_TO_CG_ML=0*v3d_lab_SEG_LENGTH

/PROX_TO_CG_AP=0*v3d_lab_SEG_LENGTH

/IXX=0

/IYY=0

/IZZ=0

! /AP_DIRECTION=

! /AXIAL_DIRECTION=

! /OBJFILE=

! /COLFILE=

! /DEPTH=

;

! Landmarks

Add_Landmark

/LANDMARK_NAME=v3d_lab_lat_prox

! /CALIBRATION_FILE=

! /USER_GENERATED=TRUE

! /USE_PERCENTAGE=FALSE

! /CALIBRATION_ONLY=FALSE

! /USE_TARGETS=FALSE

/SEGMENT_NAME=LAB

! /TARGET_TYPES=

! /TARGET_NAMES=

! /MCSX=0.0

/MCSY=0.001

! /MCSZ=0.0

! /LANDMARK_LOCATION=

! /REFERENCE_LOCATION_NAME=

! /REFERENCE_LOCATION_TYPE=

! /USE_REFERENCE_LOCATION=FALSE

;

Add_Landmark

/LANDMARK_NAME=v3d_lab_med_prox

! /CALIBRATION_FILE=

! /USER_GENERATED=TRUE

! /USE_PERCENTAGE=FALSE

! /CALIBRATION_ONLY=FALSE

! /USE_TARGETS=FALSE

/SEGMENT_NAME=LAB

! /TARGET_TYPES=

! /TARGET_NAMES=

! /MCSX=0.0

/MCSY=-0.001

! /MCSZ=0.0

! /LANDMARK_LOCATION=

! /REFERENCE_LOCATION_NAME=

! /REFERENCE_LOCATION_TYPE=

! /USE_REFERENCE_LOCATION=FALSE

;

Add_Landmark

/LANDMARK_NAME=v3d_lab_lat_distal

! /CALIBRATION_FILE=

! /USER_GENERATED=TRUE

! /USE_PERCENTAGE=FALSE

! /CALIBRATION_ONLY=FALSE

! /USE_TARGETS=FALSE

/SEGMENT_NAME=LAB

! /TARGET_TYPES=

! /TARGET_NAMES=

! /MCSX=0.0

/MCSY=0.001

/MCSZ=-0.001

! /LANDMARK_LOCATION=

! /REFERENCE_LOCATION_NAME=

! /REFERENCE_LOCATION_TYPE=

! /USE_REFERENCE_LOCATION=FALSE

;

Add_Landmark

/LANDMARK_NAME=v3d_lab_med_distal

! /CALIBRATION_FILE=

! /USER_GENERATED=TRUE

! /USE_PERCENTAGE=FALSE

! /CALIBRATION_ONLY=FALSE

! /USE_TARGETS=FALSE

/SEGMENT_NAME=LAB

! /TARGET_TYPES=

! /TARGET_NAMES=

! /MCSX=0.0

/MCSY=-0.001

/MCSZ=-0.001

! /LANDMARK_LOCATION=

! /REFERENCE_LOCATION_NAME=

! /REFERENCE_LOCATION_TYPE=

! /USE_REFERENCE_LOCATION=FALSE

;

**Appendix B: Visual3D and OpenSim xml Files Modifications**

As mentioned previously, the Visual3D to OpenSim process is designed to work with OpenSim in the manor outlined in the OpenSim tutorial “*Generating a Muscle-Actuated Simulation in OpenSim*” (in the OpenSim_GeneratingMuscleActuatedSimulation.pdf). To work successfully the process required that special versions of several OpenSim xml input be created. Below is the list of new OpenSim xml files and how the original files were modified:

**Visual3d_Setup_Scale.xml** (modified from subject01_Setup_Scale.xml)

- The <Scale Tool> name has been changed to Visual3D.
- **The subject <mass> and <height> needs to be customized for each subject**
  - Visual3D v6.3 and newer export the mass and height to the Setup_Scale file so this does not need to be done manually
- The <MarkerSet> is no longer required and reference to this file has been removed
- <scaling_order> is set only to *manualScale.* (Visual3D will write the manual scale factors for the segments to the Visual3d_Scale_ScaleSet.xml file.)
- The <MeasurementSet> .xml file and its corresponding .trc file are no longer needed and reference to these files have been removed
- The <ModelScaler> section has been removed since this is done by Visual3D
- The <MarkerPlacer> section has been removed since this is done by Visual3D

**Visual3d_Setup_RRA.xml** (modified from subject01_Setup_RRA.xml)

- The <CMCTool> name has been changed to Visual3d_walk1_RRA
- The <model_file> name has been changed to Visual3d_simbody.osim.
- The <initial_time> and <final_time> for RRA have been set to 0.0 and 1.0 but this will be changed by the user when operating OpenSim to match the desired time range for RRA
- The <desired_kinematics_file> is now Visual3d_SIMM_input.mot. This is the name of the file that is written by Visual3D containing the kinematics.
- The <external_loads_file> is now Visual3d_SIMM_grf.mot. This is the name of the file that is written by Visual3D containing the fore platform information.
- The <external_loads_model_kinematics_file> is now Visual3d_SIMM_input.mot. This is the name of the file that is written by Visual3D containing the fore platform information.
- An “external_loads_file” tag has been added to point RRA to the Visual3d_SIMM_input_grf.xml file.

**Visual3d_Setup_CMC.xml** (modified from subject01_Setup_CMC.xml)

- The <CMCTool> name has been changed to Visual3d_walk1.
- The <model_file> has been changed to Visual3d_simbody_adjusted.osim.
- The <initial_time> and <final_time> for CMC have been set to 0.0 and 1.0 but this will be changed by the user when operating OpenSim to match the desired time range for CMC
- The <desired_kinematics_file> has been changed to: ResultsRRA/Visual3d_walk1_RRA_kinemtics_q.sto.
- The <external_loads_file> is now Visual3d_SIMM_grf.mot. This is the name of the file that is written by Visual3D containing the fore platform information.
- The <external_loads_model_kinematics_file> is now Visual3d_SIMM_input.mot. This is the name of the file that is written by Visual3d containing the fore platform information.
- An “external_loads_file” tag has been added to point RRA to the Visual3d_SIMM_input_grf.xml file.

**Visual3D_Scale_ScaleSet** (modified from subject01_Scale_ScaleSet.xml)

This file must be present so Visual3D can write the manual scale factors used for the pelvis, thigh and tibia segments.

**Files No longer Required**

Visual3D does manual scaling on all segments and does its own IK, thus many of the OpenSim files related to the Marker Set, Scaling and IK are no longer required.
