# Supplementary material for: Effect of altered gluteus maximus strength on the magnitude and direction of hip joint contact forces during simulations of gait
Source: PLoS One. 2025 Jun 23;20(6):e0324451. doi: 10.1371/journal.pone.0324451 (PMC12184943; doi:10.1371/journal.pone.0324451)
Supplement: S11 Files — (ZIP) [file pone.0324451.s011.zip › V3D_Osim_Files/OpenSim_GeneratingMuscleActuatedSimulation.pdf]

Generating a Muscle-Actuated Simulation in OpenSim

Frank C. Anderson, Eran Guendelman, Scott Delp
Neuromuscular Biomechanics Laboratory
Stanford University

Overview

This document introduces the tools that enable you to generate muscle-actuated forward dynamic simulations of movement using OpenSim. You will generate a simulation by running through the workflow depicted in Fig. 1 on an example dataset.

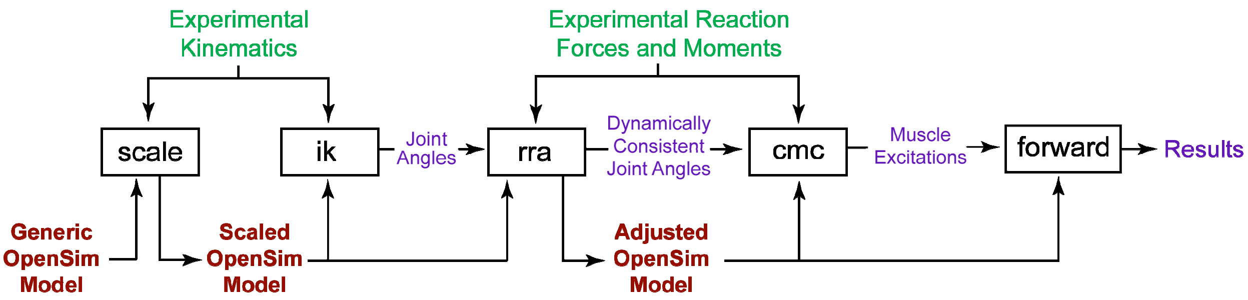

Fig. 1: Workflow for generating a simulation from experimental data. OpenSim has tools for scaling a generic musculoskeletal model to the dimensions of a subject (**scale**), solving an inverse kinematics problem to find model joint angles that best reproduce the subject's motion (**ik**), reducing dynamic inconsistencies between the model and experimental data (**rra**), solving for muscle excitations that drive the model to track the experimental motion using computed muscle control (**cmc**), and running forward dynamic simulations to generate analysis results (**forward**).

The files for the example dataset are located in your OpenSim installation, which on most computers is ***C:\Program Files\OpenSim 1.0***, in the folder ***examples\Gait2354_Simbody***.

The generic musculoskeletal model used in this example has 23 degrees of freedom and is actuated by 54 musculotendon units (Fig. 2). It is a simplified version of the lower-extremity model published by Delp et al. (1990) and later modified by Darryl Thelen, PhD to include a torso and back joint based on Anderson and Pandy (1999). This model has been simplified for the purpose of running through the example quickly and has not been validated for research.

The gait data were collected by Chand John at the University of Delaware in the Neuromuscular Biomechanics Lab directed by Jill Higginson, PhD. The data include marker trajectories and reaction forces for an adult male walking at a self-selected speed on an instrumented split-belt treadmill (Fig. 3).

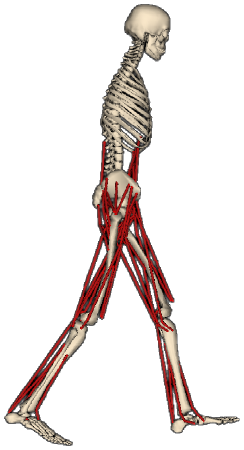

Fig. 2: Generic musculoskeletal model.

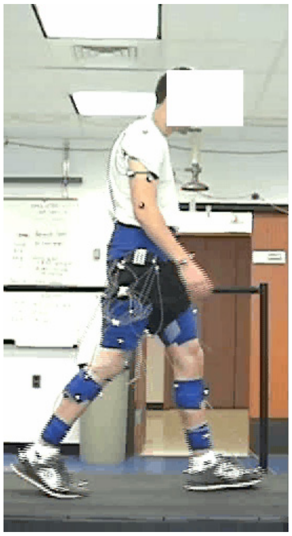

Fig. 3: Experimental setup.

The next section covers the basic steps for generating a simulation. Following that is a description of the files in the example data set, along with the basic file types you will encounter in OpenSim.

STEPS FOR GENERATING A MUSCLE-ACTUATED SIMULATION

The menu titles and option names you must select and any commands you must type or run in OpenSim appear in **bold face**. File and directory names are shown in *italic* or ***bold italic***. When executing a step, wait until execution finishes before proceeding to the next step. A step is finished when the progress bar in the lower left-hand corner of the OpenSim application window reaches 100% and then clears and goes away.

1. Make a copy of the Gait2354_Simbody folder.

a. In a Windows explorer window, find the OpenSim examples folder (e.g., ***C:\Program Files\OpenSim 1.0\examples***).

b. Make a copy of the folder ***Gait2354_Simbody***. Make sure your copy resides alongside the original in *OpenSim 1.0\examples*. This is to ensure OpenSim will be able to find the geometry files for the bones in the model.

c. Rename the folder ***My_Gait2354_Simbody***. You will step through the example in this folder so that the original folder remains unchanged.

2. Load the generic musculoskeletal model.

a. Start OpenSim if you have not already done so. To start OpenSim, go to the Windows **start** menu, select **All Programs > OpenSim 1.0 > OpenSim**.

b. In OpenSim, click the **File** menu and select **Open Model…**.

c. Find the ***examples*** folder, which is located under your OpenSim installation directory, e.g., *C:\Program Files\OpenSim 1.0*

d. Open the ***My_Gait2354_Simbody*** folder, select the file ***gait2354_simbody.osim***, and click **Open**.

3. Run the scale tool to scale the model to fit the subject.

a. In OpenSim, click the **Tools** menu and select **Scale Model…**. This will bring up the Scale Tool dialog.

b. In the lower left-hand corner of the Scale Tool dialog, click the **Settings >** and then **Load Settings...**. This will bring up a file browser.

c. In the file browser, go to the ***examples\My_Gait2354_Simbody*** folder, if you are not already there.

d. Select the file ***subject01_Setup_Scale.xml*** and click **Open**. The file *subject01_Setup_Scale.xml* contains preconfigured settings to scale the generic musculoskeletal model to the dimensions of the subject.

e. In the Scale Tool dialog, click **Run**.

f. **Close** the Scale Tool dialog.

g. A new scaled model will appear in the 3D View.

4. Run the inverse kinematics tool to solve for joint angles.

a. In OpenSim, click the **Tools** menu and select **Inverse Kinematics…**. This will bring up the Inverse Kinematics Tool dialog.

b. In the lower left-hand corner of the Inverse Kinematics Tool dialog, click the **Settings >** and then **Load Settings...**. This will bring up a file browser.

c. In the file browser, go to the ***examples\My_Gait2354_Simbody*** folder, if you are not already there.

d. Select the file ***subject01_Setup_IK.xml*** and click **Open**. The file *subject01_Setup_IK.xml* contains preconfigured settings to solve for the model joint angles that best reproduce the measured kinematics of the subject measured during a sample walking trial.

e. In the Inverse Kinematics Tool dialog, click **Run**.

f. **Close** the Inverse Kinematics Tool dialog.

g. You will see the model animate. The model virtual markers (shown in pink) should correspond closely to the experimental marker locations (shown in blue) as the animation proceeds.

h. The progress bar at the bottom right corner of the OpenSim application window will indicate the tool's progress. Wait until the bar disappears before proceeding to the next step.

5. Visualize the inverse kinematics solution in a 3D View and save it to a file.
   a. In OpenSim, open the Navigator window if it is not already open. Go to the **Window** menu and click **Navigator**.
   b. In the Navigator window, expand the scaled model **subject01** by clicking on the + sign to the right of the model name.
   c. Expand the **Motions** branch. Using your mouse, right click on the motion labeled **first trial** and select **Make Current**. The label "first trial" should be displayed in bold to indicate that it is the current motion.
   d. Using the motion slider and video controls located across the right top of the OpenSim application window, view the inverse kinematics solution. The model should walk through about one full gait cycle.
   e. Save the inverse kinematics solution to a file. Using your mouse, right click on the motion **first trial** and select **Save As…**. A save dialog will open.
   f. In the save dialog, go to the ***examples\My_Gait2354_Simbody*** folder, if you are not already there.
   g. Enter ***subject01_walk1_ik.mot*** in the **File name:** text box, and click **Save**.

6. Run the computed muscle control tool to reduce residuals.
   (RRA = Residual Reduction Algorithm (Delp et al., *in press*))
   a. In OpenSim, click the **Tools** menu and select **Computed Muscle Control…**. This will bring up the CMC Tool dialog.
   b. In the lower left-hand corner of the CMC Tool dialog, click the **Settings** > and then **Load Settings...**. This will bring up a file browser.
   c. In the file browser, go to the ***examples\My_Gait2354_Simbody*** folder, if you are not already there.
   d. Select the file ***subject01_Setup_RRA.xml*** and click **Open**. The file *subject01_Setup_RRA.xml* contains preconfigured settings to make adjustments to the model torso center of mass and to the experimental kinematics so that the residuals (i.e., the forces and moments applied to the pelvis) are smaller. Smaller residuals indicate better dynamic consistency between the experimental kinematics and ground reaction forces.
   e. In the CMC Tool dialog, click **Run**. If the **Run** button is grayed out and the **Desired kinematics** text box is filled with the color red, this indicates that the desired kinematics file *subject01_walk1_ik.mot* could not be found. Go back to step 5 above and make sure that you have saved the kinematics solution to file.
   f. **Close** the CMC Tool dialog.
   g. You will see the model animate. The motion should be very similar to the inverse kinematics solution. After the kinematics have been adjusted, a new model that has had its torso center of mass adjusted will appear in the view.

7. Close the generic musculoskeletal model.
    a. To improve the frame rate of the 3D view, close the generic musculoskeletal model. In the Navigator, using your mouse, right click on the model **3DGaitModel2354** and click **Close**.
    b. You will be asked if you would like to save settings for this model to file. Click **No**.

8. Rename the adjusted model and make sure it is the current model.
    a. You should now see two models in the Navigator labeled **subject01**. To help distinguish the two, rename the adjusted model (i.e., the last model in the Navigator). Using your mouse, right click on the adjusted model, click **Rename…**, and enter **subject01_adjusted**.
    b. If subject01_adjusted is not shown in the Navigator in bold, make it the current model. Using your mouse, right click on subject01_adjusted, and click **Make Current**.

9. Run the computed muscle control tool to solve for muscle excitations.
    a. In OpenSim, click the **Tools** menu and select **Computed Muscle Control…**. This will bring up the CMC Tool dialog.
    b. In the lower left-hand corner of the CMC Tool dialog, click the **Settings >** and then **Load Settings...**. This will bring up a file browser.
    c. In the file browser, go to the ***examples\My_Gait2354_Simbody*** folder, if you are not already there.
    d. Select the file ***subject01_Setup_CMC.xml*** and click **Open**. The file *subject01_Setup_CMC.xml* contains preconfigured settings to solve for the muscle excitations that drive the model to closely track the motion of the subject.
    e. In the CMC Tool dialog, click **Run**.
    f. **Close** the CMC Tool dialog.
    g. You will see most muscles turn blue and the model animate. The color of each muscle indicates its activation level; blue indicates no activation, and red indicates full activation.

10. Run a forward dynamic simulation to generate results.
    a. In OpenSim, click the **Tools** menu and select **Forward Dynamics…**. This will bring up the Forward Dynamics Tool dialog.
    b. In the lower left-hand corner of the Forward Tool dialog, click the **Settings >** and then **Load Settings...**. This will bring up a file browser.
    c. In the file browser, go to the ***examples\My_Gait2354_Simbody*** folder, if you area not already there.
    d. Select the file ***subject01_Setup_Forward.xml*** and click **Open**. The file *subject01_Setup_Forward.xml* contains preconfigured settings to run a forward simulation, which is now possible since muscle excitations were determined in step 9.
    e. In the Forward Dynamics Tool dialog, click **Run**.
    f. **Close** the Forward Dynamics Tool dialog.

g. You will see the model animate, and, just like while running the computed muscle control tool, the colors of the muscles will change indicating their activation levels. The forward simulation executes faster, however, because the muscle excitations are not being computed.

h. When the forward simulation completes, a number of results files are written to file in the directory ***examples\My_Gait2354_Simbody\ResultsForward***. These can be plotted in OpenSim, or using some other plotting program like Microsoft Excel or Matlab.

11. Plot the muscle force generated by the right gastrocnemius muscle.

a. In OpenSim, click the **Tools** menu and select **Plot…**. This will bring up a Plotter window.

b. In the **Curve Add** section, click **Y-Quantity** and then select **New File** at the bottom of the popup menu.

c. In the open dialog,

d. In the file browser, go to the ***examples\My_Gait2354_Simbody\ResultsForward*** folder, select the file ***subject01_walk1_Actuation_force.sto***, and click **Open**. This will bring up a dialog for selecting the data columns you want to plot.

e. In the **Select data column(s) to plot** dialog in the **Filter by pattern** text box, type **gas**. This will filter the names of all the muscle and display only those muscles that have gas in their names. Select the check box for the **med_gas_r** and click **OK**.

f. In the **Curve Add** section, click **X-Quantity**. This will bring up a dialog box with a vertical list of possible x quantities. Select **time** and click **OK**.

g. In the **Curves List** section, click **Add**. A plot showing the predicted force output of the medial gastrocnemius as a function of time is generated.

WORKFLOW INPUT, OUTPUT, AND SETTINGS FILES

In the preceding section you ran the workflow by simply loading setting that were preconfigured for each step in the workflow. Many of these settings can be adjusted from within OpenSim, while others are specified in external files referred to by the main settings file. To assist in you in configuring the workflow, this section provides a brief description of the input, output, and settings files for each of the steps in the workflow. These example files can serve as a starting template for generating muscle-actuated simulations from your own experimental data. In the diagrams and descriptions that follow, experimental data are shown in **green**; OpenSim model files (.osim) are shown in **red**; settings files are shown in **blue**; files generated by the workflow are shown in **purple**, unless it is a generated model, in which case it is shown in red like the other models. Only the output files necessary to run through the workflow are shown. Typically additional output files (e.g., results of analyses) are generated as well.

Scale

The scale tool uses a combination of measured distances between markers and manual scale factors to scale the model to the dimensions of the subject.

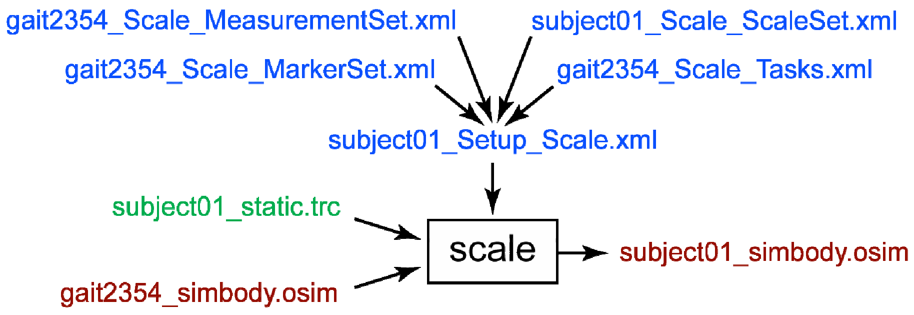

Settings Files

subject01_Setup_Scale.xml: Setup file for the scale tool. It contains settings and refers to other files that contain additional settings.

gait2354_Scale_MarkerSet.xml: Contains the set of virtual markers that are placed on the body segments of the model.

gait2354_Scale_MeasurementSet.xml: Contains pairs of experimental markers the distance between which are used to scale the generic musculoskeletal model.

subject01_Scale_ScaleSet.xml: Contains a set of manual scale factors that can be applied to the generic musculoskeletal model.

gait2354_Scale_Tasks.xml: In addition to scaling the model, the scale tool is capable of moving the virtual markers on the model so that their positions match the experimental marker locations. To do this the scale tool must position the model so that it best matches the position of the subject. This requires an inverse kinematics problem to be solved. This file contains the inverse kinematics tasks (i.e., a specification of which virtual and experimental markers should be matched up during the inverse kinematics solution) and their relative weightings.

Input

subject01_static.trc: Experimental marker trajectories for a static trial. A static trial is usually several seconds of data with the subject posed in a known static position.

gait2354_simbody.osim: OpenSim musculoskeletal model for the 23-degree-of-freedom, 54-muscle gait model. "_simbody" is appended to the file name to indicate that the Simbody dynamics engine is used compute the dynamics of the model.

Output

subject01_simbody.osim: OpenSim musculoskeletal model scaled to the dimensions of the subject.

Inverse Kinematics

The inverse kinematics tool solves for the time histories of model joint angles that best reproduce the motion of the subject as recorded in the laboratory.

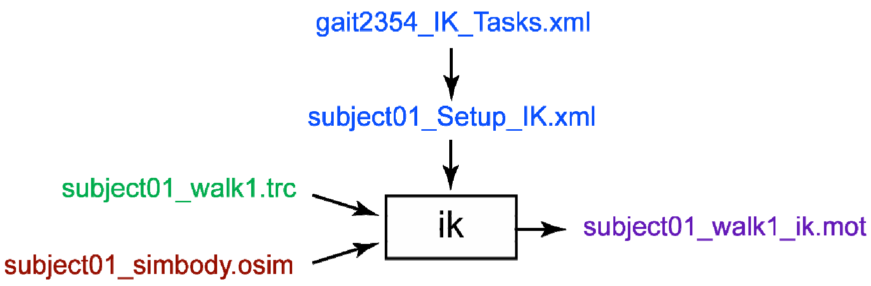

Settings Files

subject01_Setup_IK.xml: Setup file for the ik tool. It contains settings and refers to other files that contain additional settings.

gait2354_IK_Tasks.xml: This file specifies the inverse kinematics tasks for solving for the model joint angles that best reproduce the recorded subject motion. Tasks may be marker tasks in which the distance between a virtual marker on the model and an experimental marker is minimized as a sum of least squared errors. Tasks may also be coordinate tasks in which a joint angle in the model is biased toward being a specified value.

Input

subject01_walk1.trc: Experimental marker trajectories for a motion trial.

subject01_simbody.osim: OpenSim musculoskeletal model scaled to the dimensions of the subject.

Output

subject01_walk1_ik.mot: Motion file containing the time histories of joint angles that reproduce the recorded motion of the subject.

Residual Reduction

Experimental kinematics and reaction forces are frequently dynamically inconsistent. This dynamic inconsistency is reflected in the need to apply additional residual forces and moments to the model to make the model undergo the same motion as the subject. The residuals can be due to measurement errors in the kinematics and reaction forces, modeling assumptions (e.g., a rigid torso segment), or a combination of the two. The size of the residuals is a measure of the dynamic inconsistency. This step in the workflow adjusts the center of mass the model torso segment and slightly alters the kinematics solution to reduce the residuals, achieving better dynamic consistency. Residual reduction is accomplished by using the computed muscle control algorithm to track the inverse kinematics solution but at the same time penalizing large residuals (Delp et al., *in press*).

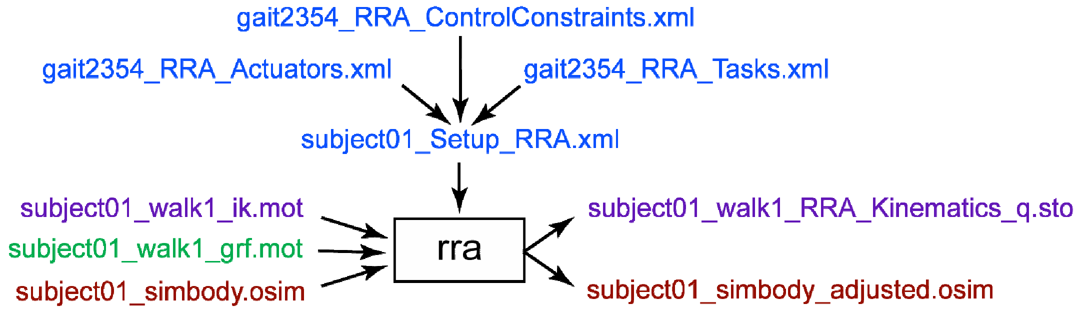

Settings Files

subject01_Setup_RRA.xml: Setup file for the rra step in the workflow. It contains settings and refers to other files that contain additional settings.

gait2354_RRA_Actuators.xml: Contains a set of idealized joint actuators, one for each generalized coordinate in the model, that are used to actuate the model replacing the muscles. Large residuals are penalized by choosing relatively small maximum force/torque values for the residual actuators.

gait2354_RRA_ControlConstraints.xml: The force/torque output of each actuator in the model is determined by the value of control variable. This file sets the maximum and minimum allowed values for the actuator controls.

gait2354_RRA_Tasks.xml: Specifies the joint angles that should be tracked by the computed muscle control algorithm. Each tracking task contains variables that specify its weight relative to other tasks, as well as gains on the position and velocity errors.

Input

subject01_walk1_ik.mot: Motion file containing the time histories of joint angles that should be tracked. This file was generated by the inverse kinematics tool.

subject01_walk1_grf.mot: Motion file containing the measured ground reaction forces that should be applied to the model during simulation.

subject01_simbody.osim: OpenSim musculoskeletal model scaled to the dimensions of the subject.

Output

subject01_walk1_RRA_Kinematics_q.sto: Storage file containing the time histories of joint angles that have been adjusted to reduce the residuals. Typical adjustments to the joint angles are small (less than a degree).

subject01_simbody_adjusted.osim: OpenSim musculoskeletal model scaled to the dimensions of the subject with a torso center of mass that has been adjusted to reduce residuals.

Computed Muscle Control

Computed muscle control solves for a set of muscle excitations that drive the musculoskeletal model to track a set of desired joint angles (Thelen and Anderson, 2006).

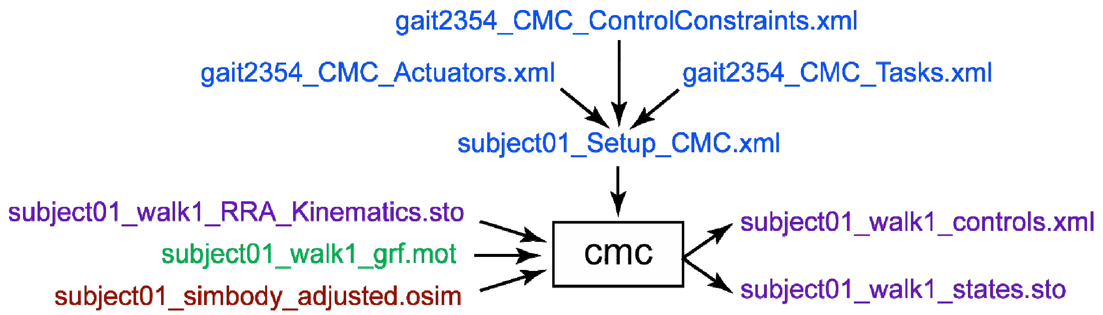

Settings Files

subject01_Setup_CMC.xml: Setup file for the computed muscle control tool. It contains settings and refers to other files that contain additional settings.

gait2354_CMC_Actuators.xml: Contains a set of actuators that supplement the muscles of the model. Six of the actuators correspond to the residual forces and moments applied to the pelvis (i.e., the base segment of the model). The remainder are reserve actuators that allow the computed muscle control algorithm to find a solution when muscles are too weak or cannot change quickly enough to track the desired motion., one for each generalized coordinate in the model, that are used to actuate the model replacing the muscles. Large residuals are penalized by choosing relatively small maximum force/torque values for the residual actuators.

gait2354_CMC_ControlConstraints.xml: The force/torque output of each actuator in the model is determined by the value of control variable. This file sets the maximum and minimum allowed values for the actuator controls.

gait2354_CMC_Tasks.xml: Specifies the joint angles that should be tracked by the computed muscle control algorithm. Each tracking task contains variables that specify its weight relative to other tasks, as well as gains on the position and velocity errors.

Input

subject01_walk1_RRA_Kinematics_q.sto: Motion file containing the time histories of joint angles that should be tracked. This file was generated by the residual reduction step.

subject01_walk1_grf.mot: Motion file containing the measured ground reaction forces that should be applied to the model during simulation.

subject01_simbody_adjusted.osim: OpenSim musculoskeletal model scaled to the dimensions of the subject with a torso center of mass that has been adjusted to reduce residuals.

Output

subject01_walk1_controls.xml: Contains the time histories of muscle excitations computed by the computed muscle control tool.

subject01_walk1_states.sto: Contains the time histories of model states computed by the computed muscle control tool, including joint angles, joint speeds, muscle activations, and more.

Forward Dynamic Simulation

Once a set of muscle excitations has been computed, it is possible to execute a muscle-actuated forward dynamic simulation. This allows you to generate analysis results or perturb the model to ask "what-if" questions.

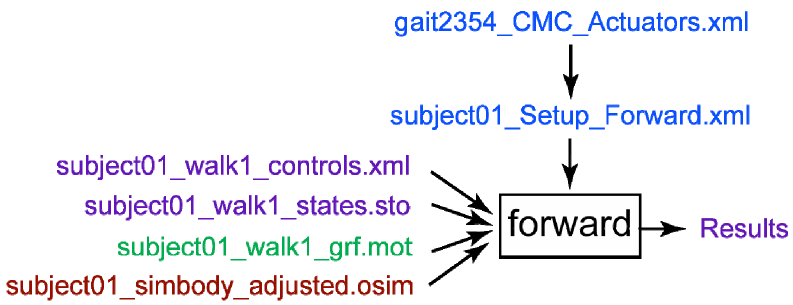

Settings Files

subject01_Setup_Forward.xml: Setup file for the forward dynamics tool. It contains settings and refers to other files that contain additional settings.

gait2354_CMC_Actuators.xml: Contains a set of actuators that supplement the muscles of the model. Refer to the previous section on computed muscle control for more details. These

actuators must be included in the forward simulation so that the computed muscle control solution can be reproduced.

Input

subject01_walk1_controls.xml: Contains the time histories of muscle excitations computed by the computed muscle control tool. These controls were computed by the computed muscle control tool.

subject01_walk1_states.sto: Contains the time histories of model states including joint angles, joint speeds, muscle activations, and more. These states were computed by the computed muscle control tool, and are used by the forward dynamics tool to set the initial states of the model for forward integration.

subject01_walk1_grf.mot: Motion file containing the measured ground reaction forces that should be applied to the model during simulation.

subject01_simbody_adjusted.osim: OpenSim musculoskeletal model scaled to the dimensions of the subject with a torso center of mass that has been adjusted to reduce residuals.

Output

Results: Analyses can be added to the forward dynamics tool so that results can be written to file. These analyses are specified in the setup file (**subject01_Setup_Forward.xml**).

OpenSim File Types

OpenSim Model File (*.osim)

OpenSim model files contain the actuators, bodies, joints, coordinates, and speeds of a model. The format is xml (extensible markup language) and can be viewed and edited using **Edit > File (.osim,.xml)** in OpenSim or using most text editors. It can also be viewed using most web browsers.

OpenSim Property File (*.xml)

OpenSim property files contain, for example, settings for tools or additional data for models including marker sets, actuator sets, or control values. The format is xml (extensible markup language) and can be viewed and edited using **Edit > File (.osim,.xml)** in OpenSim or using most text editors. It can also be viewed using most web browsers.

Motion File (*.mot)

SIMM motion files from MusculoGraphics, Inc. are text files that contain time-sequence data commonly encountered in motion capture systems. The data are arranged in columns with each column representing, for example, a joint angle or a component of the ground reaction force. Each row in a motion file corresponds to a different time. Time spacing between rows is assumed to be uniform.

Storage File (*.sto)

Storage files are very similar to motion files except they have the added flexibility of handling non-uniform time spacing between rows.

REFERENCES

1. Anderson F.C., Pandy M.G. A dynamic optimization solution for vertical jumping in three dimensions. *Computer Methods in Biomechanics and Biomedical Engineering*, vol. 2, pp. 201-231, 1999.

2. Anderson F.C., Pandy M.G. Dynamic optimization of human walking. *Journal of Biomechanical Engineering*, vol. 123, pp. 381-390, 2001.

3. Delp, S.L., Anderson, F.C., Arnold, A. S., Loan, P., Habib, A., John, C., Thelen, D.G. OpenSim: Open-source software to create and analyze dynamic simulations of movement. *IEEE Transactions on Biomedical Engineering*, (in press).

4. Delp, S.L., Loan, J.P., Hoy, M.G., Zajac, F.E., Topp E.L., Rosen, J.M. An interactive graphics-based model of the lower extremity to study orthopaedic surgical procedures. *IEEE Transactions on Biomedical Engineering*, vol. 37, pp. 757-767, 1990.

5. Thelen D.G., Anderson F.C. Using computed muscle control to generate dynamic simulations of human walking from experimental data. *Journal of Biomechanics*, vol. 39, pp. 1107-1115.
